# Comparative Analysis of Radiosensitizers for K-RAS Mutant Rectal Cancers

**DOI:** 10.1371/journal.pone.0082982

**Published:** 2013-12-12

**Authors:** Laura B. Kleiman, Angela M. Krebs, Stephen Y. Kim, Theodore S. Hong, Kevin M. Haigis

**Affiliations:** 1 Molecular Pathology Unit, Center for Cancer Research and Center for Systems Biology, Massachusetts General Hospital, Charlestown, Massachusetts, United States of America; 2 Institute of Molecular Medicine and Cell Research, Albert Ludwigs University Freiburg, Germany; 3 Department of Radiation Oncology, Massachusetts General Hospital, Boston, Massachusetts, United States of America; Indiana University School of Medicine, United States of America

## Abstract

Approximately 40% of rectal cancers harbor activating K-RAS mutations, and these mutations are associated with poor clinical response to chemoradiotherapy. We aimed to identify small molecule inhibitors (SMIs) that synergize with ionizing radiation (IR) (“radiosensitizers”) that could be incorporated into current treatment strategies for locally advanced rectal cancers (LARCs) expressing mutant K-RAS. We first optimized a high-throughput assay for measuring individual and combined effects of SMIs and IR that produces similar results to the gold standard colony formation assay. Using this screening platform and K-RAS mutant rectal cancer cell lines, we tested SMIs targeting diverse signaling pathways for radiosensitizing activity and then evaluated our top hits in follow-up experiments. The two most potent radiosensitizers were the Chk1/2 inhibitor AZD7762 and the PI3K/mTOR inhibitor BEZ235. The chemotherapeutic agent 5-fluorouracil (5-FU), which is used to treat LARC, synergized with AZD7762 and enhanced radiosensitization by AZD7762. This study is the first to compare different SMIs in combination with IR for the treatment of K-RAS mutant rectal cancer, and our findings suggest that Chk1/2 inhibitors should be evaluated in new clinical trials for LARC.

## Introduction

 An estimated 1.2 million people worldwide are diagnosed with colorectal cancer (CRC) each year, and around 600,000 people die from the disease [1]. More effective treatment options are urgently needed. Low-grade CRCs can be cured with surgery alone, whereas later stage cancers are additionally treated with some combination of chemotherapy, IR, and targeted therapies, depending on the anatomic site and staging of the tumor. Targeted therapies (SMIs and monoclonal antibodies (mAbs)) affect signaling pathways aberrantly activated in cancer cells and are slowly making their way into the clinic for the treatment of various cancers, either as monotherapies or else to improve responses to standard of care treatments. Three such drugs (all mAbs) are approved to treat metastatic CRC: cetuximab and panitumumab, which inhibit the epidermal growth factor receptor (EGFR; a member of the ErbB family of receptor tyrosine kinases) and show benefit only for K-RAS wild-type cancers, and bevacizumab, which inhibits the angiogenesis-promoting vascular endothelial growth factor (VEGF) [2]. These mAbs have thus far not demonstrated benefit for locally advanced disease [3,4], and no targeted therapies are approved for non-metastatic CRC.

 Because of their anatomic location, surgical resections are more challenging for rectal cancer compared to colon cancer, and therefore there is a greater risk of local recurrence [5,6]. Pre-operative radiotherapy decreases the local recurrence rate and is used in combination with chemotherapy to treat LARC [7,8]. Nevertheless, only 10% of these patients achieve a pathologically complete response (pCR) and one-third die within 5 years [9,10]. Strategies to improve response aim to increase the cytotoxic effect of IR to the tumor cells without similarly affecting normal tissue, in order to minimize treatment side effects. Identification of chemoradiosensitizing drugs is particularly pertinent for the ~40% of rectal cancer patients that harbor K-RAS mutations [8]. Mutant K-RAS has been extensively linked to radioresistance in human cancer cell lines [11–17]. Moreover, the response of LARC patients to chemoradiotherapy is highly variable, with some patients exhibiting pCR and others a minimal response. K-RAS mutations are more common in patients with non-pCR [18], which is associated with decreased disease-free survival [10].

K-RAS is a small GTPase that functions downstream of cell surface receptors, such as EGFR, and switches between an inactive, GDP-bound state and an active, GTP-bound state [19]. GTP-bound K-RAS activates various signaling cascades, including the canonical Raf-MEK-ERK (MAPK) and PI3K-Akt-mTOR pathways, to regulate cellular processes such as proliferation and survival. Mutations in K-RAS are most frequently found at codons 12 and 13 and compromise GTP hydrolysis stimulated by GTPase-activating proteins (GAPs), resulting in hyperactive K-RAS and uncontrolled proliferation [19].

IR produces different DNA lesions, with the most prominent being DNA double-strand breaks (DSBs), and often arrests cells at the G1-S or G2-M transition of the cell cycle to allow for DNA repair [20]. If there are substantial or irreparable lesions, cells may die through apoptosis or necrosis or undergo cellular senescence [21]. Radiotherapy may be improved by modulating DNA repair, cell cycle checkpoints, or signal transduction pathways such as the MAPK or PI3K pathways [22,23]. Nevertheless, the optimal strategy for incorporating targeted therapies into treatment regimens is unclear. In this study, we optimized a high-throughput radiosensitization screen for rectal cancer cell lines and identified radiosensitizing drugs for K-RAS mutant rectal cancers.

## Materials and Methods

### Cell culture and drug solutions

DLD-1 and HCT116 colon cancer cells were obtained from the Vogelstein laboratory (Johns Hopkins Kimmel Cancer Center, Baltimore, MD). Rectal and pancreatic cancer cell lines were obtained from the Center for Molecular Therapeutics (Massachusetts General Hospital, Boston, MA). DLD-1 and HCT116 cells were cultured in DMEM supplemented with 10% FBS. Rectal and pancreatic cancer cell lines were cultured in DMEM/F-12 supplemented with 5% FBS. See Table S1 for cell line information and Table S2 for drug information.

### Drug and radiation treatments

The day after seeding cells, the media was replaced with media containing vehicle or drug, and IR was applied 2 hours later with a JL Shepherd Mark I Model 25 irradiator with a Cs-137 source. Sham irradiated (0 Gy) controls were treated exactly the same as irradiated samples except that no radiation was applied. No edge wells of 96-well plates were used for analysis. Drug-containing media was replaced every other day, but radiosensitization results were similar when drug was not replaced.

### Colony formation assay

Cells were seeded in 6-well plates such that each well contained at least 20 colonies that were minimally touching after 2-3 weeks. Cells were fixed and stained for 30 minutes with a mixture of 6% glutaraldehyde and 0.5% crystal violet in distilled water. Plates were scanned and colonies with at least 50 cells were counted using ImageJ. The surviving fraction (SF) following drug and IR treatments was defined as: (plating efficiency x number of colonies formed) / (number of cells plated), where the plating efficiency for a given cell line was defined for the corresponding control treatment as: (number of colonies) / (number of cells seeded).

### CyQUANT

The CyQUANT Direct Cell Proliferation Assay (Life Technologies, Molecular Probes) was performed in 96-well plates in accordance with the manufacturer’s instructions, except that the CyQUANT Direct Nucleic Acid Stain was used at a final concentration of 1:1000 and the CyQUANT Direct Background Suppressor I at 1:200, and the incubation period was 30 minutes. Fluorescence was measured with a SpectraMax M5 fluorescence plate reader (Ex/Em=485/538nm). Background signal from wells with media but no cells was subtracted out.

### Hoechst staining and analysis

Cells in 96-well plates were fixed for 10 minutes in 4% paraformaldehyde and stained with Hoechst nucleic acid stain 33342 (Molecular Probes) diluted 1:40,000 in PBS for 30 minutes. For each well, images were obtained with a Zeiss Axio Observer Z1 microscope at four locations spaced 100µm apart and subsequently analyzed with the CellProfiler2 Cell Image Analysis software (www.cellprofiler.org). The average nuclei count for the four images of each well was used for further analysis.

### Cell counting

Cells were seeded in 6- or 12-well plates and at end of the experiment were detached using trypsin/EDTA and collected in growth media. The cell suspension was diluted 1:1 in 0.2% trypan blue and the cell concentration and viability were assessed using a Nexcelom Cellometer Auto T4 cell counter. The number of live cells was used for further analysis.

### CellTiter-Glo

The CellTiter-Glo Luminescent Cell Viability Assay (Promega) was performed in 96-well plates in accordance with the manufacturer’s instructions, except that 1/4 of the recommended amount of CellTiter-Glo Reagent was used per well. Background signal from wells with media but no cells was subtracted out.

### Calculation of surviving fractions

SFs are described as the fraction of cells remaining following treatment. The averages of triplicate experiments were plotted and the error bars represent the standard deviations. Data were normalized to vehicle plus sham IR. For plots of data following IR treatment, to account for the effects of drug alone, drug plus IR was additionally normalized to drug plus sham IR for each drug concentration (represented by a dashed line at the value 1). A SF for drug plus IR less than the SF for vehicle plus IR suggests synergy. Student’s t-tests were used to evaluate whether the mean SFs for treatments and controls were significantly different. p-values ≤ 0.05 for drug plus IR treatment compared to vehicle control plus IR are indicated by asterisks in the figures.

### Western blotting

Western blotting was performed using RIPA lysis buffer and standard methods. For cleaved PARP measurements, floating cells were collected each time the media was changed and at the end of the experiment, and after lysis combined with lysate from the adherent cells. Membranes were incubated with primary antibodies diluted in Odyssey Blocking Buffer overnight at 4°C, washed in PBS containing 0.1% Tween-20, and incubated for 1 hour at room temperature with secondary antibodies diluted 1:10,000 in Odyssey Blocking Buffer. Membranes were scanned using a LI-COR Odyssey infrared imager and Odyssey 2.1 software was used for quantification. See Table S3 for a list of the antibodies used.

### Cell cycle profiling

Cells were seeded in 6-well plates and following treatment were washed in PBS, trypsinized, and collected in media. All subsequent steps were performed on ice or in a centrifuge set to 4°C. Cells were centrifuged at 1500 rpm for 5 minutes and washed in PBS, and then were resuspended in Mg2+- and Ca2+-free PBS. 95% EtOH was added dropwise while vortexing on low speed, and the cells were stored at -20°C until use. Cells were then centrifuged for 5 minutes at 2000 rpm, washed twice in PBS, resuspended in 1mg/mL RNaseA, and then transferred to FACS tubes. Propidium iodide (PI) diluted in PBS was added to a final PI concentration of 1µg/mL. Cells were kept in the dark until analyzed on a LSR II quad-laser cytometer running FACSDiva software (BD Immunocytometry). FlowJo version 7.6 was used to analyze the data with the Dean-Jett-Fox (DJF) mathematical model.

## Results

### Optimization of the high-throughput assay (HTA)

 The effects of radiation on cultured cell lines are typically assessed via a colony formation assay (CFA). This approach is not amenable to high-throughput analysis, however, because it is time consuming, expensive, and requires significant optimization for each cell line and treatment. As such, our initial goal was to optimize a HTA for measuring responses to drugs and IR. First, we generated a reference dataset with the CFA, where cells were seeded in 6-well plates, a range of IR doses were applied on the subsequent day, and the number of colonies was assessed 2 weeks later. Next, cells were seeded in 96-well plates, IR was applied on the subsequent day, and the number of viable cells was assessed by the CyQUANT assay after 3 to 8 days (Figure S1). The 1 week time point for 96-well plates produced similar results to the CFA. 

We found that treatment of some rectal cancer cell lines with IR and/or SMIs caused nuclei and cells to enlarge and flatten. In these cases, CyQUANT and other common proliferation assays that are based on DNA content (e.g. Syto-60) produced an inaccurate readout of cell number that clearly disagreed with what we observed by visual inspection. This effect was particularly striking for IR-treated RCM-1 cells (Figure S2A), but not for SW837 cells. We decided to stain nuclei in the 96-well plates with Hoechst, image the plates, and then estimate the number of cells in each well by segmenting nuclei with the CellProfiler software. This assay produced qualitatively similar results to cell counting 1 week post-IR (low-throughput) and the CFA for both cell lines (Figure S2B). The protocol (“HTA”) used for the radiosensitization screen is shown in Figure S3.

### Radiosensitization screen

 The drugs selected for the screen affect diverse signaling pathways, such as those with a known role in radiosensitization, as well as those thought to be hyperactivated in cancer but without an established role in radiation response. We included drugs in oncology pipelines at the major pharmaceutical companies with promising pre-clinical or clinical data. In some cases, multiple drugs targeting the same pathway were included to compare their potencies and to address the role of off-target effects. The final selection of drugs comprised 28 SMIs (Table 1). We used drug concentrations ranging from ~10nM to 1µM, since these are typically clinically achievable concentrations. IR doses ranged from 2 Gy to 8 Gy since LARC patients often receive fractionated doses of 1.8 Gy IR, but sometimes receive higher doses.

**Table 1:** Drugs evaluated as single agents and combined with radiation in the screen.

**Drug**	**Primary target**
Gefitinib	EGFR
AZD8931	pan-ErbB
GDC-0941	PI3K
BKM120	pan-PI3K
BEZ235	PI3K/mTOR
GDC-0980	PI3K/mTOR
Perifosine	AKT/mTOR
Sorafenib	Raf/VEGFR/etc.
PLX4032	Raf (V600E)
Raf265	Raf/VEGFR
CI-1040	MEK
AZD6244	MEK
GSK1120212	MEK
PD325901	MEK
SP600125	JNK II
LY2228820	p38
Dovitinib	FGFR/PDGFR
Abt888	PARP
AT-406	IAPs
AZD1480	JAK1/2
PD0332991	CDK4/6
AZD7762	Chk1/2
KU-55933	ATM
Vorinostat	HDAC
AUY922	Hsp90
AZD1152	Aurora kinase
Sunitinib	multiple targets
Midostaurin	multiple targets

**Figure 1:**
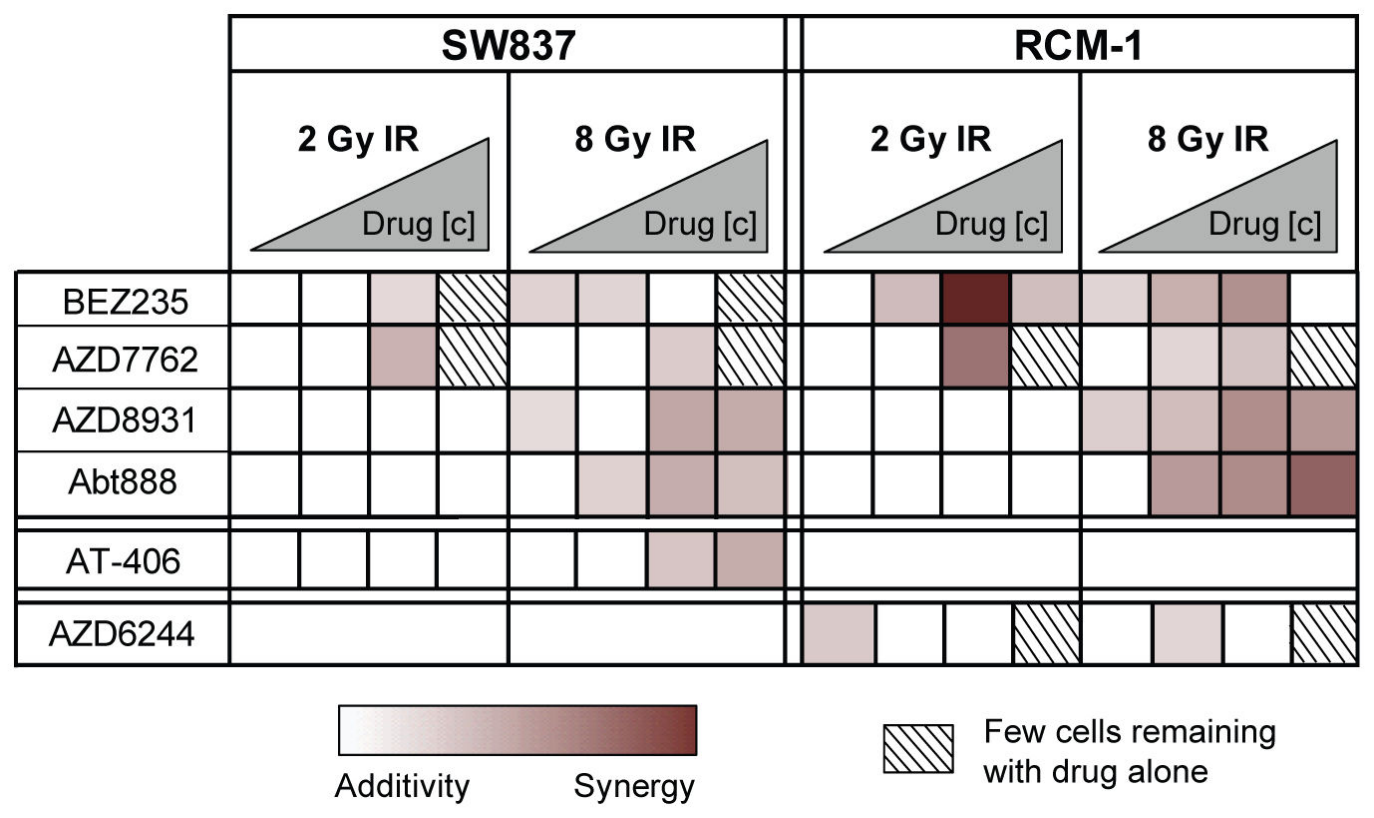
Heat map summarizing Bliss independence (BI) values for the strongest radiosensitizers. BI values correspond to (F_Cexp_ - F_Cobs_ ) x 100 and were calculated based on the raw data from the screen depicted in Figure S4. Drug concentrations ranged from 10nM to 1.25µM. Radiosensitization was not considered if the surviving fraction (SF) following drug treatment alone was less than 0.125, since a synergistic effect of the combination with IR may not be detectable. Although CI-1040, Raf265, and GDC-0980 were slightly radiosensitizing, they were not included in follow-up studies since other inhibitors of the same pathways (AZD6244 and BEZ235) exhibited more potent synergy.

 The K-RAS mutant rectal cancer cell lines SW837 and RCM-1 were used for the screen, and the results are shown in Figure S4. The SMIs had a wide range of potencies, with MEK inhibitors generally being the strongest on their own (Figure S5A). We used two measures to quantitatively characterize radiosensitization. First, we calculated the degree of radiosensitization by comparing the differences in SFs resulting from SMI treatment with and without IR (e.g. Figure S5B). Second, we used Bliss independence (BI) to determine whether the effects are antagonistic, additive, or synergistic. BI assumes that the activities of the SMI and IR are mutually exclusive. The expected combined effect (F_Cexp_) is the fractional product of the effects of the individual treatments. The difference between F_Cexp_ and the observed combined effect (F_Cobs_) determines whether the two treatments are antagonistic (F_Cexp_ < F_Cobs_), additive (F_Cexp_ = F_Cobs_), or synergistic (F_Cexp_ > F_Cobs_). No antagonism (negative BI value) was detected between any SMI and IR. BI values for the six strongest radiosensitizers are illustrated in Figure 1. Only the PI3K/mTOR inhibitor BEZ235 and the checkpoint kinases 1 and 2 (Chk1/2) inhibitor AZD7762 synergized with 2 Gy IR in both cell lines. 

For the five inhibitors used that target the PI3K/Akt/mTOR pathway, SW837 cells were more sensitive to drug alone, whereas RCM-1 cells were more strongly radiosensitized. All of these drugs at least slightly radiosensitized RCM-1 cells at 8 Gy IR, but only BEZ235 was strongly radiosensitizing at 2 Gy IR. On the other hand, all four inhibitors targeting MEK were more potent on their own in RCM-1 cells and showed a trend toward slight radiosensitization of RCM-1 but not SW837 cells.

### BEZ235 and AZD7762 radiosensitize

Radiosensitization was confirmed for BEZ235 and AZD7762 by CFA (Figure S6). These SMIs radiosensitized over a range of IR doses as low as 0.5 or 1 Gy (Figure S7). Since 1.8 Gy IR is typically administered for five consecutive days for multiple weeks during the course of treatment for LARC, we asked whether the SMIs are still radiosensitizing with fractionated doses of IR. We first evaluated the effects of applying 2 Gy IR on four consecutive days, but this left too few cells remaining for radiosensitization analysis (Figure S8A-B). Nevertheless, our results suggested that drugs that are radiosensitizing with one application of IR are also radiosensitizing using this protocol (data not shown). To be able to evaluate the effects of consecutive days of IR, we applied 0.5 or 1 Gy on four consecutive days. We detected similar radiosensitization by BEZ235 and AZD7762 as when IR was applied once (Figure S8C-D).

**Figure 2:**
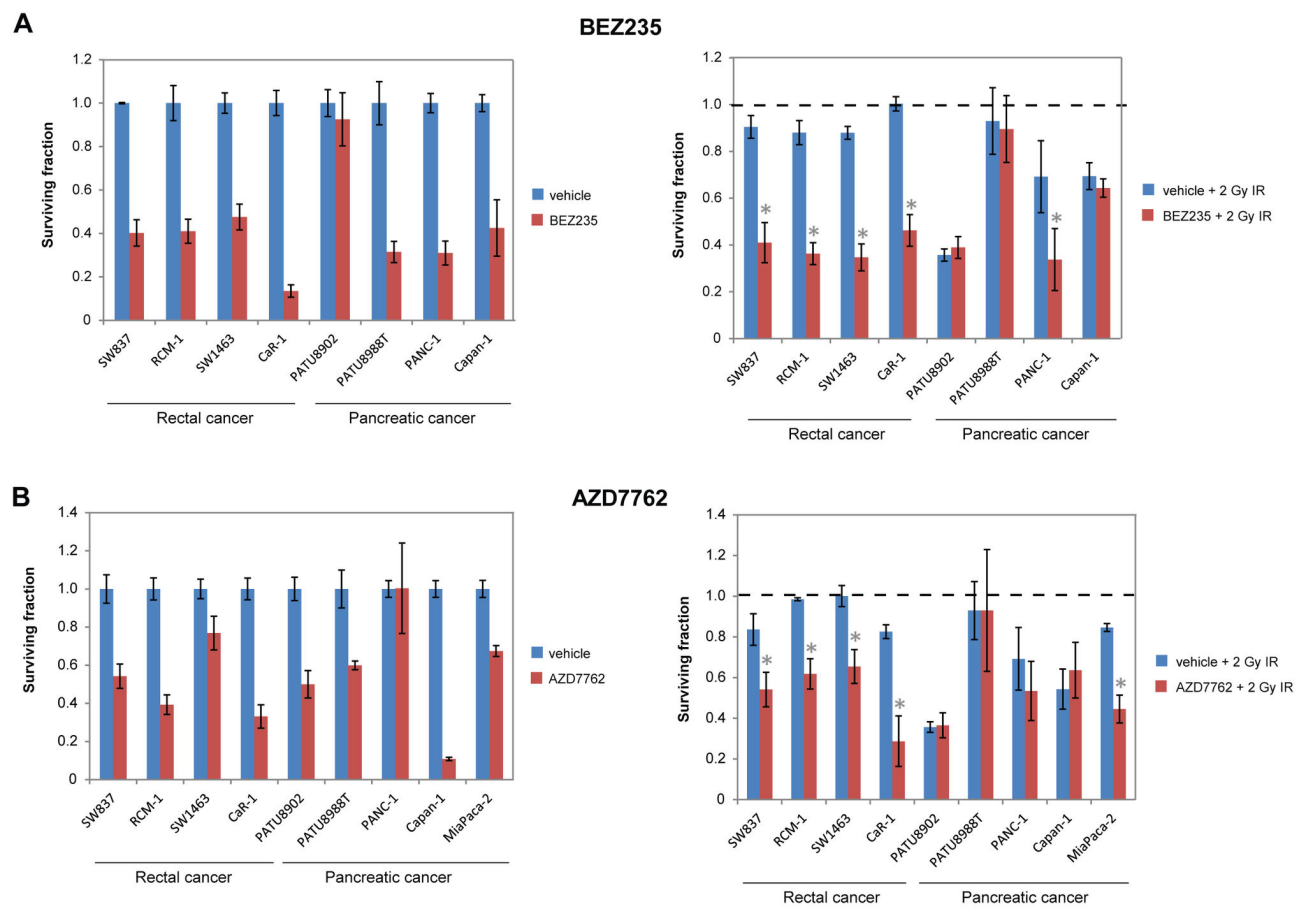
All rectal cancer but only some pancreatic cancer cell lines are radiosensitized by BEZ235 and AZD7762. Effects of BEZ235 (A) or 250nM AZD7762 (B) 1 week post-IR. Different concentrations of BEZ235 were used depending on the sensitivity of each cell line to BEZ235 treatment in the absence of IR (the pancreatic lines were more sensitive to BEZ235): 250nM for SW837, RCM-1, and SW1463 cells, 50nM for CaR-1 and Capan-1 cells, 25nM for PANC-1 cells, and 1nM for PATU8902 and PATU8988T cells. The HTA was used for the rectal cancer cell lines and Capan-1 cells, and cell counting was used for the remaining lines. *Left*, Data are normalized to vehicle plus sham IR treatment (effects of the SMIs in the absence of IR). *Right*, Data are normalized to corresponding non-irradiated controls. p-values ≤ 0.05 for IR plus SMI treatment compared to IR plus vehicle control are indicated by asterisks.

**Figure 3:**
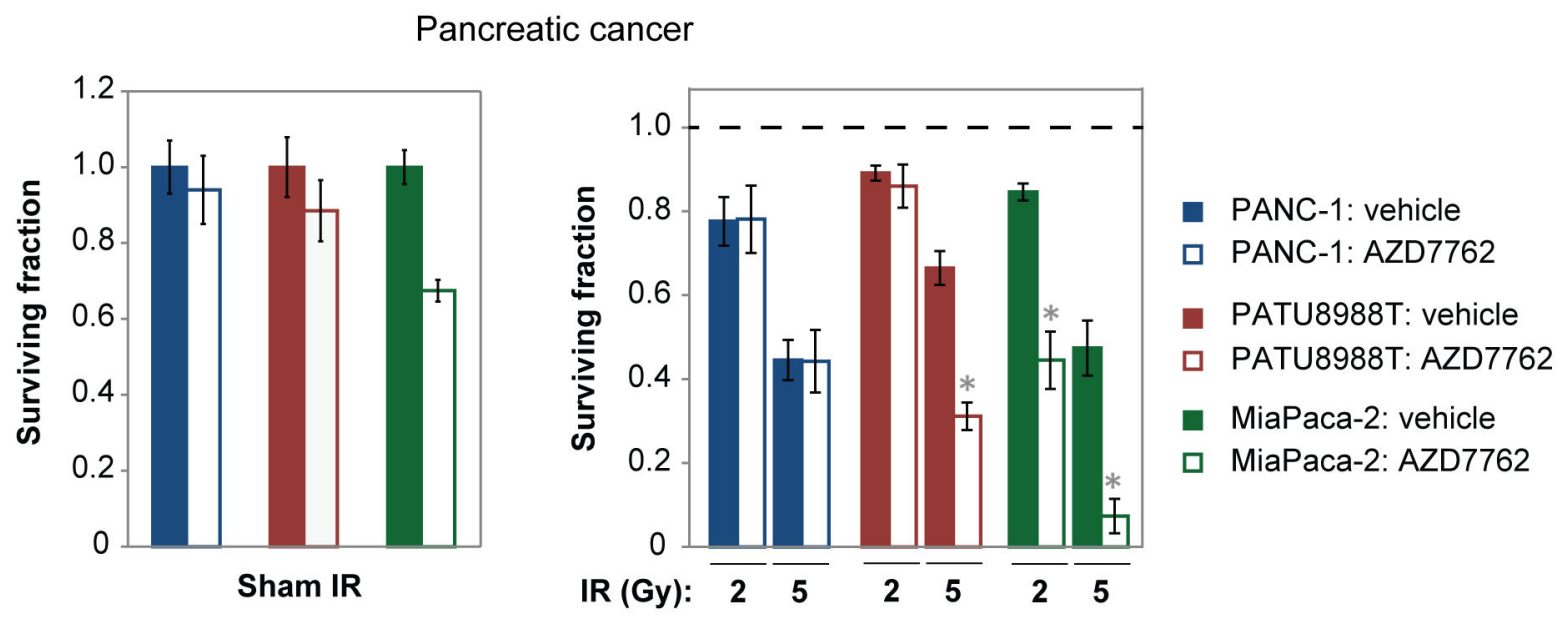
Pancreatic cancer cell lines exhibit variability in response to combination treatment of 250nM AZD7762 and IR. The CellTiter-Glo assay was performed 1 week post-IR. *Left*, Data are normalized to vehicle plus sham IR treatment (effects of the SMIs in the absence of IR). *Right*, Data are normalized to corresponding non-irradiated controls; solid bars represent the effects of IR alone. p-values ≤ 0.05 for IR plus SMI treatment compared to IR plus vehicle control are indicated by asterisks.

Most pancreatic cancers harbor K-RAS mutations [19], and these tumors are often treated with radiotherapy. To investigate the scope of our findings, follow-up studies were performed with the only two other established rectal cancer cell lines (SW1463 and CaR-1) and with pancreatic cancer cell lines. All of these lines harbor K-RAS mutations except for CaR-1 cells. BEZ235 strongly radiosensitized all rectal cancer cell lines but only weakly radiosensitized one pancreatic cancer cell line (PANC-1) at 2 Gy IR (Figure 2A). AZD7762 radiosensitized all rectal cancer cell lines but only the MiaPaca-2 pancreatic cancer cells at 2 Gy IR (Figure 2B). AZD7762 has been evaluated primarily for pancreatic cancer in combination with gemcitabine, and MiaPaca-2 cells are most often used in these pre-clinical studies [24,25]. Radiosensitization was also detected in PATU8988T cells at 5 Gy IR (Figure 3), a dose that is being evaluated in clinical trials. Nevertheless, pancreatic cancer cell lines exhibited a more variable response to AZD7762 in the presence of IR compared to rectal cancer cell lines, and our data suggest that BEZ235 and AZD7762 may more universally radiosensitize rectal cancers.

### 5-FU synergizes with AZD7762 and enhances radiosensitization

**Figure 4:**
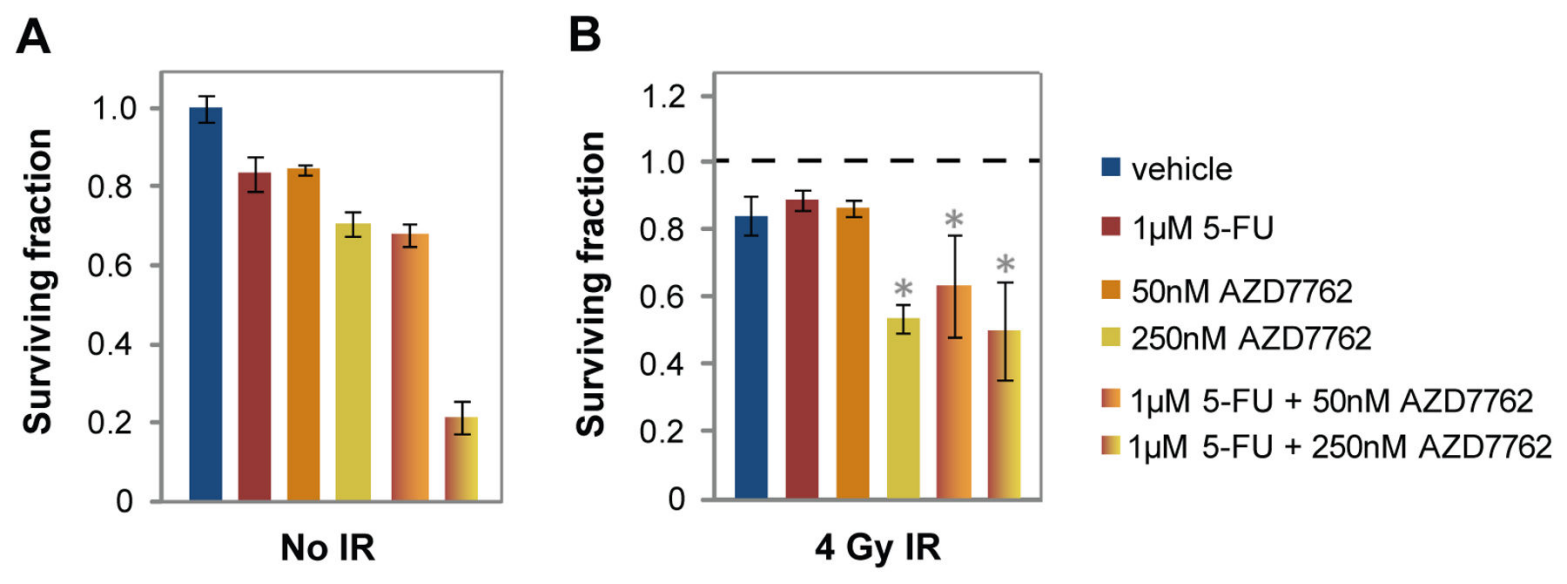
The chemotherapeutic agent 5-FU synergizes with AZD7762 and enhances radiosensitization by AZD7762. HTA results for SW837 cells are shown. See Figure S10 for data on RCM-1 cells and BEZ235 treatment. (A) Data are normalized to vehicle plus sham IR. (B) Data are normalized to corresponding non-irradiated controls. p-values ≤ 0.05 for IR plus SMI treatment compared to IR plus vehicle control are indicated by asterisks.

A SMI incorporated into a clinical trial for LARC patients would be administered concomitantly with IR and 5-FU-based chemotherapy [3]. Anti-metabolites such as 5-FU and gemcitabine inhibit DNA replication and result in an S-phase arrest. AZD7762 treatment was synergistic with 5-FU in the rectal cancer cell lines in the absence of IR (Figure 4A (see data for 250nM AZD7762) and S9), but no synergy was detected for BEZ235 and 5-FU (Figure S10). Furthermore, 5-FU enhanced radiosensitization by AZD7762, but not by BEZ235 (Figure 4B (see data for 50nM AZD7762) and S10). We therefore focused on AZD7762 as the most promising chemoradiosensitizer.

### Combination treatment of AZD7762 and IR promotes DNA damage and apoptosis

IR-induced DSBs are detected by the serine/threonine protein kinases ataxia telangiectasia mutated (ATM) and ATM and Rad 3-related kinase (ATR), which phosphorylate many intracellular substrates, including Chk1/2, H2AX and p53 [26]. Chk1 and Chk2 are serine/threonine kinases that play essential roles in the response to DSBs by maintaining the S- and G2-phase checkpoints and regulating homologous recombination repair [26,27]. ATR phosphorylates Chk1 on S317 and S345, which catalytically activates Chk1 and leads to autophosphorylation on S296, and ATM phosphorylates Chk2 on T68, leading to Chk2 homodimerization and autophosphorylation on T383, T387 and S516 [28,29]. Chk1 kinase inhibitors increase phosphorylation of Chk1 S345 due to ATR activation and diminished dephosphorylation by PP2A [30,31]. Similarly, Chk2 kinase inhibitors increase phosphorylation of Chk2 T68, which is ATM-dependent and regulated by multiple protein phosphatases [32].

Our results in the rectal cancer cell lines are consistent with these well-established effects of Chk1/2 inhibitors and AZD7762. Chk1 and Chk2 activity (Chk1 pS296 and Chk2 pS516) were inhibited by AZD7762 in the presence and absence of IR (Figures S11A and S12A), indicating that the drug effectively blocked its targets. Phosphorylation of Chk1 and Chk2 on ATM/ATR-mediated sites (Chk1 pS345 and Chk2 pT68) increased following AZD7762 treatment (Figures S11B and S12B). Phosphorylation of Chk1 on S345 is known to promote Chk1 proteolytic degradation [33], and we found that AZD7762 decreased Chk1 levels (Figure S11C).

**Figure 5:**
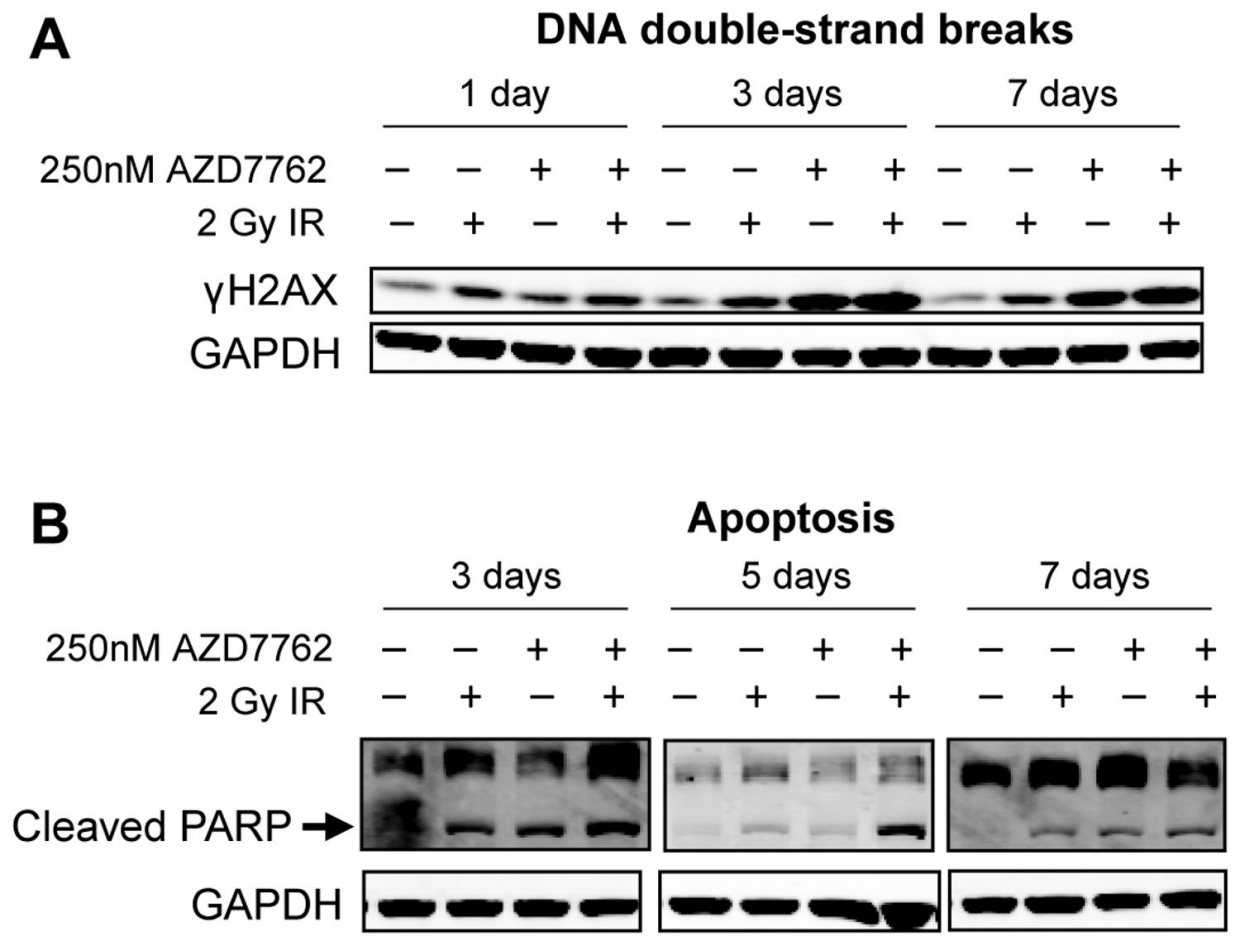
Combination treatment of AZD7762 and IR inhibits DNA repair and induces apoptosis. Western blotting results for RCM-1 cells are shown. See Figure S14 for the quantification and data on SW837 and SW1463 cells. The number of days post-IR is indicated. (A) DNA DSBs as indicated by γH2AX levels. (B) Apoptosis as indicated by cleaved PARP levels.

Cancer cells often lose their G1 checkpoint, for example through mutation of the tumor suppressor p53, and are more dependent on the G2 checkpoint and Chk1/2 activity [22]. Chk1/2 depletion or inhibition, particularly in p53-deficient cells, abrogates the G2 checkpoint induced by genotoxic agents and prevents the repair of DNA, eventually leading to mitotic catastrophe and apoptosis or senescence [20,28]. Lack of DNA repair is commonly detected by prolonged phosphorylation of the histone variant H2AX on S139, which is rapidly phosphorylated by ATM, ATR or DNA-PK in response to DSBs [34]. Indeed, we found that AZD7762 treatment abrogated the IR-induced G2 arrest (Figure S13) and led to increased phosphorylation of H2AX (γH2AX) and apoptosis in the rectal cancer cell lines (Figures 5 and S14).

### AZD7762 is a more potent radiosensitizer than other ATM-Chk1/2 inhibitors

**Figure 6:**
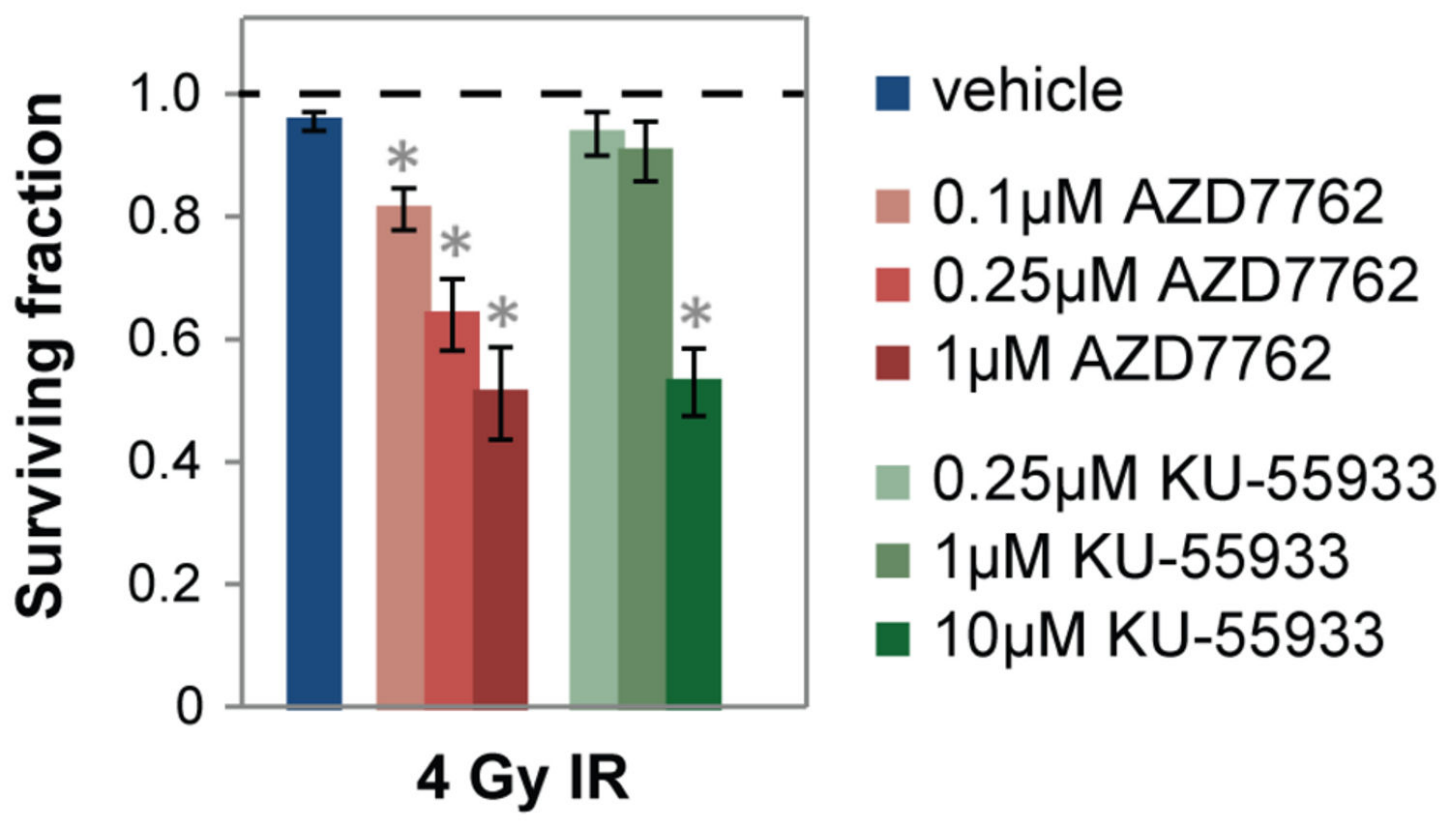
AZD7762 is a more potent radiosensitizer than the ATM inhibitor KU-55933. The CellTiter-Glo assay was performed with SW837 cells 1 week post-IR. Data are normalized to corresponding non-irradiated controls. p-values ≤ 0.05 for IR plus SMI treatment compared to IR plus vehicle control are indicated by asterisks.

**Figure 7:**
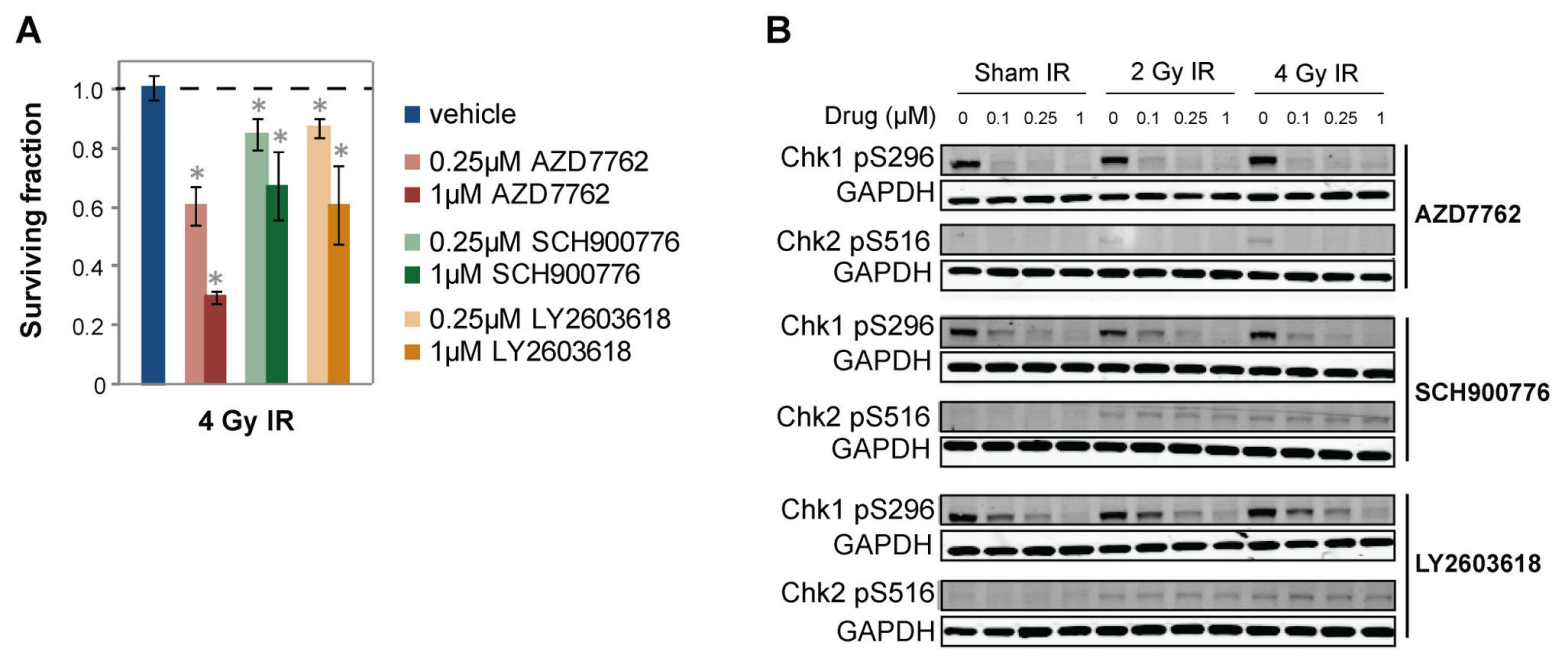
AZD7762 radiosensitizes and inhibits Chk1 activity at a 10-fold lower concentration than two Chk1-specific inhibitors. (A) The CyQUANT assay was performed with SW837 cells 1 week post-IR. SCH900776 and LY2603618 are Chk1 inhibitors. Data are normalized to corresponding non-irradiated controls. p-values ≤ 0.05 for IR plus SMI treatment compared to IR plus vehicle control are indicated by asterisks. (B) SW837 cells were treated with vehicle or SMI two hours prior to IR, and protein lysates were collected three hours post-IR. Chk1 and Chk2 autophosphorylation sites were measured by Western blotting.

We found that other drugs targeting ATM-Chk1/2 are not as good as AZD7762 at radiosensitizing rectal cancer cell lines. AZD7762 radiosensitized at around a 40-fold lower concentration than the ATM inhibitor KU-55933 (Figure 6). Several Chk1 and Chk2 inhibitors are in pre-clinical development and early clinical trials [26]. We compared AZD7762 to two others that are commercially available (SCH900776 and LY2603618) [35]. AZD7762 is similarly potent against Chk1 and Chk2 in vitro (IC_50_ = 5nM and <10nM, respectively), whereas SCH900776 is selective for Chk1 (IC_50_ = 3nM) over Chk2 (IC_50_ = 1.5μM), and LY2603618 is reported to be a selective Chk1 inhibitor but there are no published data [20]. AZD7762 radiosensitized and inhibited Chk1 activity at a 10-fold lower concentration than SCH900776 and LY2603618 (Figure 7), even though at least AZD7762 and SCH900776 have similar in vitro binding affinities for Chk1. 

## Discussion

The aim of this study was to identify new treatment options for LARC patients with K-RAS mutations by optimizing a high-throughput assay and screening a panel of SMIs targeting diverse signaling pathways for radiosensitization. Our underlying assumption for this work was that a SMI with a synergistic effect on cancer cells when combined with radiation therapy is more desirable than one that has a highly cytotoxic effect on its own. We identified six SMIs that radiosensitize K-RAS mutant rectal cancer cells: BEZ235 (PI3K/mTOR inhibitor), AZD7762 (Chk1/2 inhibitor), AZD8931 (pan-ErbB receptor inhibitor), AT-406 (inhibitor of apoptosis protein (IAP) family inhibitor), AZD6244 (MEK inhibitor), and Abt888 (PARP inhibitor). Although other inhibitors of ErbB receptors and anti-apoptotic proteins have been shown to be radiosensitizing [23], AZD8931 and AT-406 have not previously been reported to be radiosensitizers. BEZ235 and AZD7762 were the most potent radiosensitizers and we focused on these two drugs.

BEZ235 has been shown to radiosensitize tumors of various tissues, including glioblastomas, fibrosarcomas, and NCSLC cell lines harboring K-RAS mutations [36–38]. BEZ235 also normalizes tumor vasculature in xenograft experiments, leading to improved tumor perfusion, oxygenation, and responses to radiotherapy [36]. Different mechanisms have been proposed to explain the radiosensitizing effect seen with BEZ235, even within the same cell line (H460 cells): abrogation of IR-induced G_2_ arrest and apoptosis [37], and increased G_2_ arrest and senescence [39]. Common to all papers describing a radiosensitizing effect of BEZ235 is delayed repair of DNA damage. Here, we show for the first time that BEZ235 acts as a potent radiosensitizer of rectal and pancreatic cancer cell lines. Cells with PIK3CA activating mutations or loss of the PTEN tumor suppressor gene are more sensitive to BEZ235 than cells lacking these mutations [40], and the high sensitivity of PATU8902 and PATU8988T cells to BEZ235 may be explained by their PTEN deficiency (Table S1). Inhibition of PI3K and mTOR by BEZ235 appears to be a promising therapeutic approach, especially in combination with IR for rectal cancer. There have been no clinical trials with BEZ235 for CRC or combined with IR (www.clinicaltrials.gov).

AZD7762 has been shown to radiosensitize various cancer cell lines in vitro and in xenograft models by abrogating the G2 checkpoint and inhibiting DNA repair. AZD7762 has primarily been evaluated for pancreatic cancer, but has also been shown to radiosensitize prostate, lung, breast, and colon cancer cells [24,25,41–43]. Normal human fibroblasts and small intestine epithelial cells are not radiosensitized by AZD7762 [25,42].

No previous studies have compared radiosensitization by AZD7762 and other inhibitors, and we found that it is a better radiosensitizer of rectal cancer cells than inhibitors of other proteins involved in the response to genotoxic agents, as well as other inhibitors of the ATM-Chk1/2 pathway. AZD7762 has markedly different effects compared to the checkpoint kinase inhibitors SCH900776 and LY2603618 in that it is the only one that decreases viability on its own at 1µM (via DSBs and apoptosis; data not shown) and it is the strongest radiosensitizer. The simplest explanation for these differences is that AZD7762 inhibits Chk2 while the other two drugs do not, and that Chk2 plays an important role in radiosensitizing rectal cancer cells. However, many studies (primarily based on siRNA knockdown) have suggested that chemo- and radiosensitization are mediated by Chk1 and to a much lesser extent Chk2, and that the effects of AZD7762 are due to its inhibition of Chk1 [20,24,44–46]. It is unclear whether Chk2-specific inhibitors possess significant chemo- and radiosensitizing activity [47–49]. Furthermore, we show that AZD7762 is a stronger inhibitor of Chk1 than SCH900776 and LY2603618. Thus, the stronger radiosensitization by AZD7762 is likely due to its enhanced effects on Chk1, and, to a lesser extent, its inhibition of Chk2.

We found that AZD7762 synergized with 5-FU and that 5-FU enhanced radiosensitization by AZD7762. 5-FU normally confers an S-phase arrest through activation of Chk1, and Chk1 depletion abrogates this arrest by stabilizing Cdc25A to resume DNA synthesis, resulting in the accumulation of DSBs and apoptosis [50]. Similar to 5-FU, gemcitabine activates Chk1 and inhibits DNA synthesis, and the combination of gemcitabine and AZD7762 increases DSBs and enhances cytotoxicity [24,44,51,52]. While AZD7762 had not previously been tested with 5-FU, gemcitabine has been shown to sensitize cells to IR in the presence of AZD7762 by accelerating progression through S phase and abrogating the G2 checkpoint, further increasing the amount of DNA damage [24]. 

AZD7762 treatment causes mitotic catastrophe and apoptotic cell death [35]. Cell death that follows mitotic catastrophe is delayed, usually occurring 2–6 days post-IR, which is consistent with the apoptosis detected in our study (Figure S14B). Mitotic catastrophe is an oncosuppressive mechanism that results from aberrant mitosis and often precedes IR-induced cell death or senescence [21,53]. Mitotic catastrophe is a common effect of IR treatment and Chk1/2 depletion or inhibition, especially in p53-deficient cells [42,54,55], and occurs when cells fail cytokinesis and enter G1 with tetraploid DNA content, becoming giant polyploid cells with abnormal nuclei [21]. We observed enlarged SW1463 nuclei following individual AZD7762 and IR treatments, and even larger nuclei with the combination treatment (Figure S15), suggesting more mitotic catastrophe.

No correlations were detected between radiosensitization and K-RAS mutational status or K-RAS dependency (Figure 2 and Table S1). Although a wild-type K-RAS rectal cancer cell line (CaR-1) was also radiosensitized by BEZ235 and AZD7762, it is possible that K-RAS is hyperactivated by a mechanism other than mutation in these cells. Nevertheless, the mechanism of action of the radiosensitization is likely independent of K-RAS. Approximately 65% of rectal cancers harbor p53 mutations [8], and all four of the rectal cancer cell lines used in this study are mutant for p53. Based on the known roles of Chk1/2 and p53 in the DNA damage response pathway and the preferential effects of Chk1/2 knockdown and inhibitors (including AZD7762) in cells with deficient versus proficient p53 [25,42], rectal cancers with compromised p53 may be more readily radiosensitized by AZD7762. Yet slight radiosensitization by Chk1/2 inhibitors has been detected in cancer cells with wild-type p53, suggesting that clinical trials should not yet be restricted to patients with p53 mutations [20,28]. Interestingly, K-RAS mutations, but not p53 mutations, are correlated with non-pCR to chemoradiotherapy, and concurrent K-RAS and p53 mutations (20% of LARCs) is an even stronger predictor of non-pCR [18].

In this study, we identified Chk1/2 inhibitors as a promising new therapeutic approach for LARC in combination with IR. In addition, the use of Chk1/2 inhibitors for colon cancer and metastatic rectal cancer in combination with 5-FU warrants further investigation. Selective Chk1/2 inhibitors have not yet been evaluated in clinical trials for CRC or in combination with 5-FU or IR (www.clinicaltrials.gov).

## Supporting Information

Figure S1 **Optimization of the duration of the 96-well plate assay based on comparison to the colony formation assay (CFA).** The CyQUANT assay performed 1 week post-IR with DLD-1 (cell line 1) and HCT116 (cell line 2) cells, two K-RAS mutant colon cancer cell lines, produced qualitatively similar results to the CFA. *Left*, Cells were fixed and stained 2 weeks post-IR and the number of colonies was assessed. *Right*, Days indicated refer to time post-IR. For each time point, cells were seeded such that control wells were ~75% confluent at the time of analysis.
(PDF)

Figure S2 **Optimization of the high-throughput measurement assay for rectal cancer cell lines.** (A) Example Hoechst images 1 week post-IR. Fewer RCM-1 cells remain following treatment with 8 Gy IR and their nuclei are larger compared to cells treated with sham IR. (B) Hoechst is more accurate than CyQUANT for measuring the response of RCM-1 cells to IR. Cell counting, CyQUANT and Hoechst were performed 1 week post-IR whereas the CFA was performed 2 weeks post-IR. All plots are normalized to sham IR treatment.
(PDF)

Figure S3 **Protocol used for the radiosensitization screen.** Cells were treated with SMIs for 2 hours prior to irradiation.
(PDF)

Figure S4 **Heat map summarizing results from the screen performed with 28 SMIs and two K-RAS mutant rectal cancer cell lines.** Surviving fractions (SFs) were normalized to vehicle plus sham IR treatment.
(PDF)

Figure S5 **Results from the screen.** (A) The effect of each SMI (250nM) in the absence of IR for SW837 and RCM-1 cells is plotted. Data are normalized to vehicle plus sham IR (dashed line). (B) The degree of radiosensitization for each SMI (250nM) in the presence of IR (2 Gy) is plotted. The degree of radiosensitization was calculated by dividing the product of the individual effects of SMI and IR by the combined effect. Data are normalized to vehicle plus sham IR (dashed line).
(PDF)

Figure S6 **Radiosensitization of RCM-1 cells by AZD7762 (**A**) and BEZ235 (**B**) as measured by CFA.**
(PDF)

Figure S7 **HTA performed with SW837 and RCM-1 cells for AZD7762 (A) or BEZ235 (B) and different doses of IR.**
(PDF)

Figure S8 **Radiosensitization with IR applied on consecutive days.** (A) Modifications to the HTA protocol for applying IR on consecutive days. (B) 2 Gy IR applied on four consecutive days compared to 2 or 8 Gy IR applied once. (C)-(D) 0.5 or 1 Gy IR applied on four consecutive days with or without AZD7762 (C) or BEZ235 (D) compared to various doses of IR applied once. Appropriate sham irradiated controls were included.
(PDF)

Figure S9 **AZD7762 and 5-FU treatments are synergistic.** The HTA was performed and nuclei were stained with Hoechst.
(PDF)

Figure S10 **HTA results for the combination of 5-FU, IR and AZD7762 (**A**) or BEZ235 (**B**).** Synergy between AZD7762 and 5-FU was detected for RCM-1 cells at 150nM AZD7762 (Figure S9), where there is less of an effect of AZD7762 alone.
(PDF)

Figure S11 **Chk1 is inhibited by AZD7762 in rectal cancer cell lines.** The time indicated is post-IR treatment (i.e. cells were exposed to AZD7762 for 4 hours for the “2 hrs” time point). (A) Decreased phosphorylation of a Chk1 autophosphorylation site (S296) by 250nM AZD7762. (B) Increased phosphorylation of Chk1 S345, which is mediated by ATM and ATR and targets Chk1 for degradation. (C) Decreased total Chk1 levels.
(PDF)

Figure S12 **Chk2 is inhibited by AZD7762 in rectal cancer cell lines.** The time indicated is post-IR treatment. (A) IR-induced phosphorylation of a Chk2 autophosphorylation site (S516) is inhibited by AZD7762. *Right*, quantification by background subtraction, normalization to GAPDH, and normalization to control treatment. (B) Phosphorylation of Chk2 site (T68) that is mediated by ATM and ATR. 4 Gy IR was used.
(PDF)

Figure S13 **IR-induced G2 arrest is abrogated following treatment with AZD7762.** Cell cycle profiling results indicate the percent of cells in different phases of the cell cycle. Cells were treated with AZD7762 for two hours prior to IR, and the analysis was performed after 24 hours.
(PDF)

Figure S14 **Combination treatment with AZD7762 and IR results in increased DNA damage and induction of apoptosis.** (A) DSBs as indicated by γH2AX levels. *Right*, quantification by background subtraction, normalization to GAPDH, and normalization to control treatment. (B) Apoptosis as indicated by cleaved PARP levels. *Right*, quantification by background subtraction and normalization to GAPDH. Days post-IR are indicated.
(PDF)

Figure S15 **The HTA was performed with SW1463 cells and nuclei were stained with Hoechst.** Note that nuclei stain very heterogeneously with Hoechst, especially in the image of AZD7762 treatment alone.
(PDF)

Table S1 **Mutational status and K-RAS dependency of the human cell lines used in this study.**
(PDF)

Table S2 **Drugs used in this study.**
(PDF)

Table S3 **Antibodies used in this study.**
(PDF)
