# Supplementary figures and images for: Comparative Analysis of Radiosensitizers for K-RAS Mutant Rectal Cancers
Source: PLoS One. 2013 Dec 12;8(12):e82982. doi: 10.1371/journal.pone.0082982 (PMC3861465; doi:10.1371/journal.pone.0082982)

Figure S1

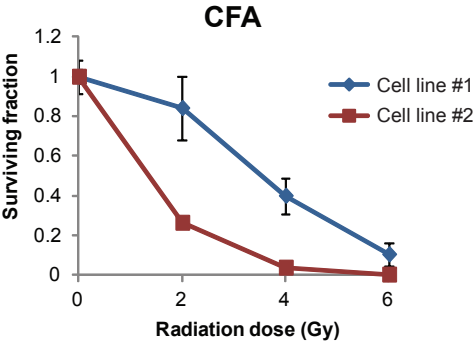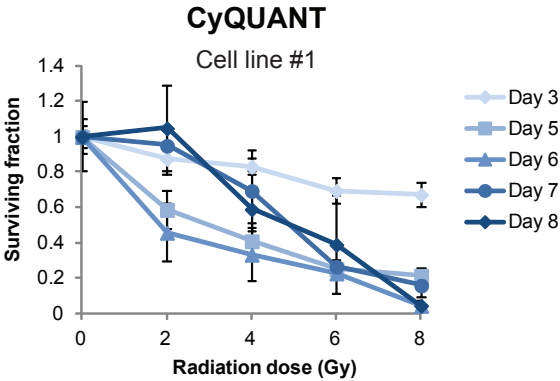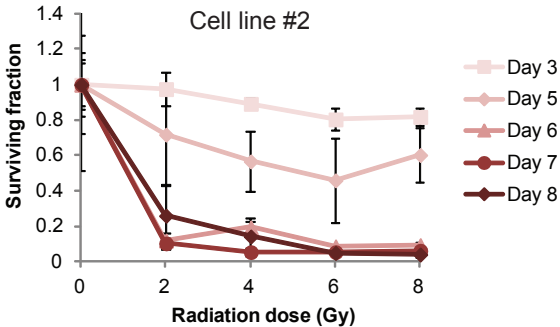

Supplement: Figure S1 — Optimization of the duration of the 96-well plate assay based on comparison to the colony formation assay (CFA). The CyQUANT assay performed 1 week post-IR with DLD-1 (cell line 1) and HCT116 (cell line 2) cells, two K-RAS mutant colon cancer cell lines, produced qualitatively similar results to the CFA. Left, Cells were fixed and stained 2 weeks post-IR and the number of colonies was assessed. Right, Days indicated refer to time post-IR. For each time point, cells were seeded such that control wells were ~75% confluent at the time of analysis. (PDF) [file pone.0082982.s001.pdf]

Figure S2

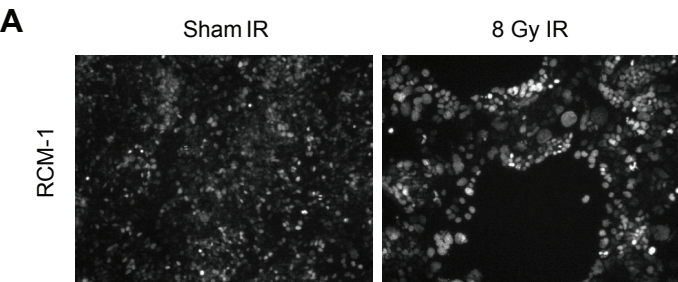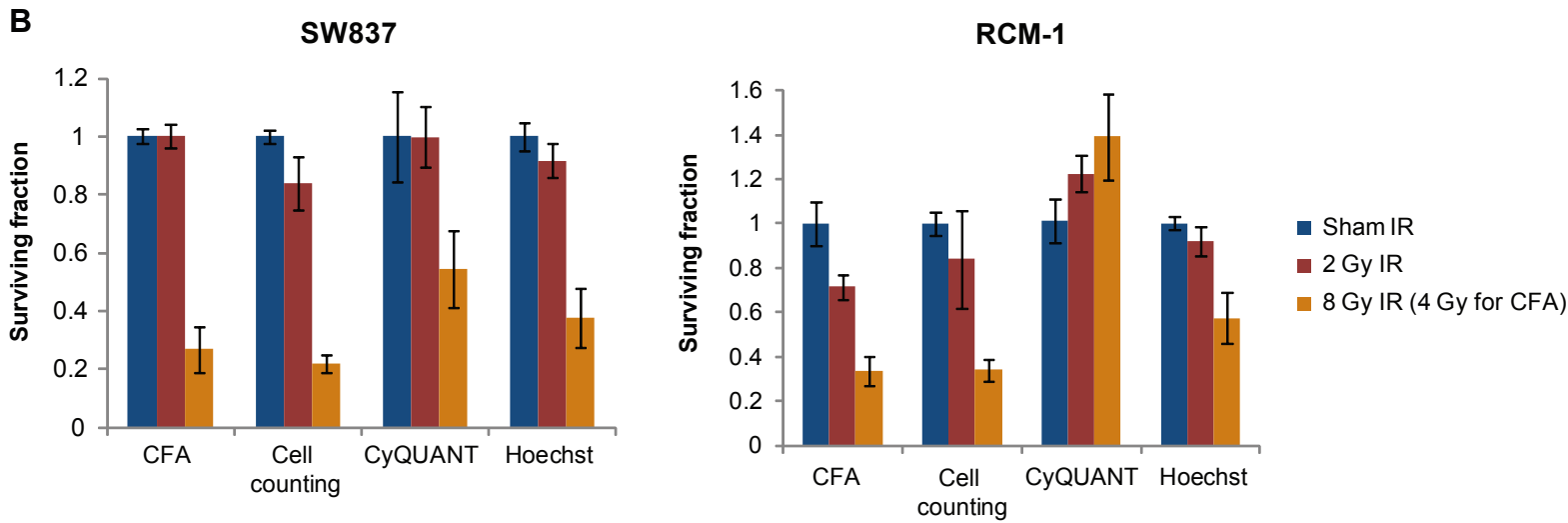

Supplement: Figure S2 — Optimization of the high-throughput measurement assay for rectal cancer cell lines. (A) Example Hoechst images 1 week post-IR. Fewer RCM-1 cells remain following treatment with 8 Gy IR and their nuclei are larger compared to cells treated with sham IR. (B) Hoechst is more accurate than CyQUANT for measuring the response of RCM-1 cells to IR. Cell counting, CyQUANT and Hoechst were performed 1 week post-IR whereas the CFA was performed 2 weeks post-IR. All plots are normalized to sham IR treatment. (PDF) [file pone.0082982.s002.pdf]

Figure S3

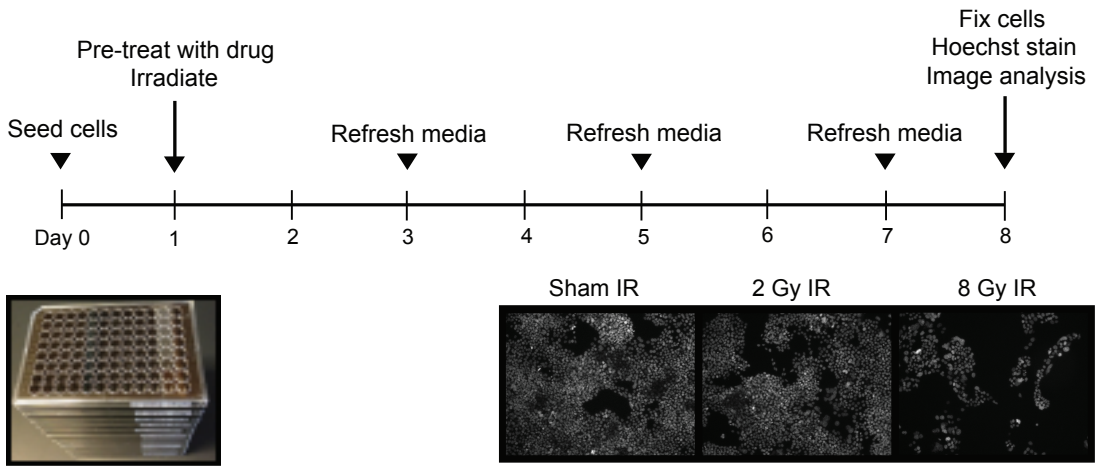

Supplement: Figure S3 — Protocol used for the radiosensitization screen. Cells were treated with SMIs for 2 hours prior to irradiation. (PDF) [file pone.0082982.s003.pdf]

Figure S4

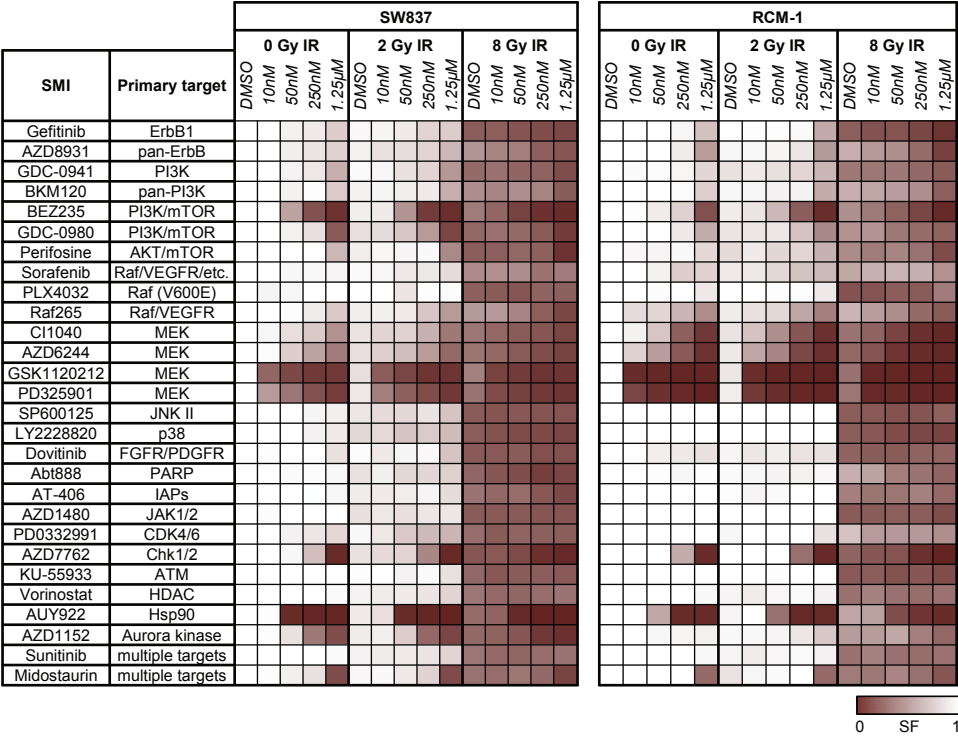

Supplement: Figure S4 — Heat map summarizing results from the screen performed with 28 SMIs and two K-RAS mutant rectal cancer cell lines. Surviving fractions (SFs) were normalized to vehicle plus sham IR treatment. (PDF) [file pone.0082982.s004.pdf]

Figure S5

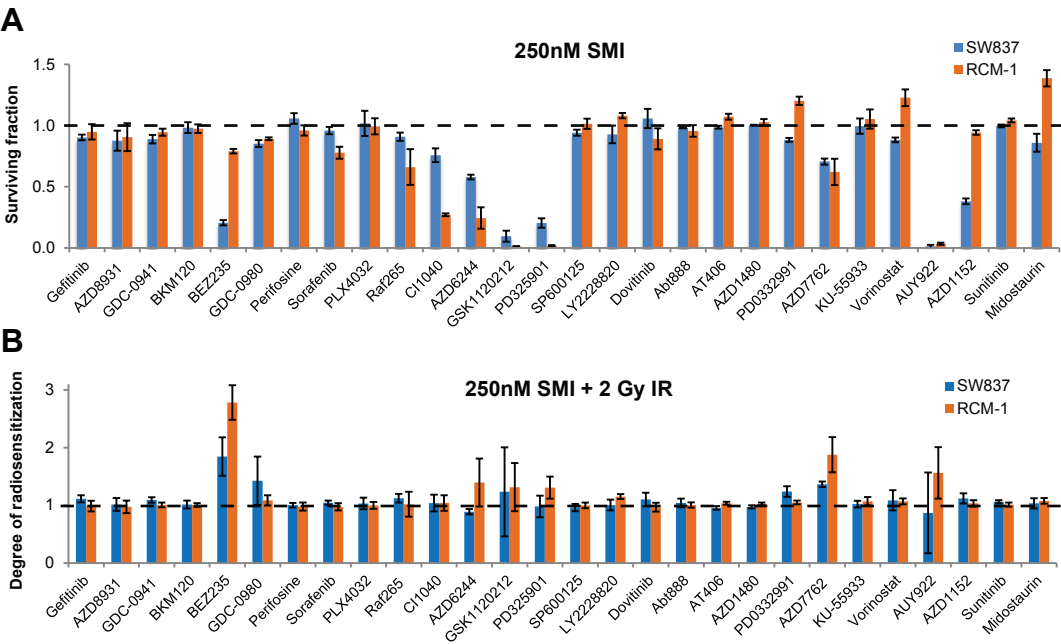

Supplement: Figure S5 — Results from the screen. (A) The effect of each SMI (250nM) in the absence of IR for SW837 and RCM-1 cells is plotted. Data are normalized to vehicle plus sham IR (dashed line). (B) The degree of radiosensitization for each SMI (250nM) in the presence of IR (2 Gy) is plotted. The degree of radiosensitization was calculated by dividing the product of the individual effects of SMI and IR by the combined effect. Data are normalized to vehicle plus sham IR (dashed line). (PDF) [file pone.0082982.s005.pdf]

Figure S6

A

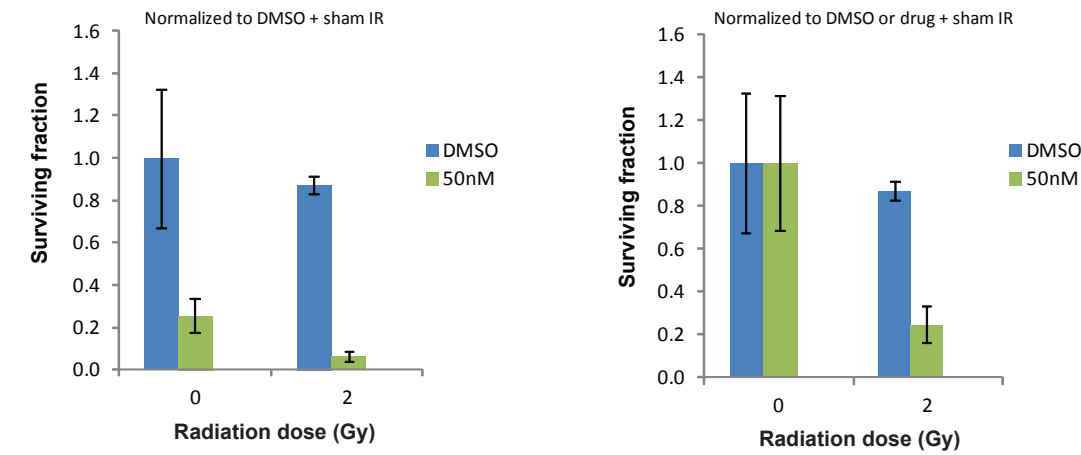

B

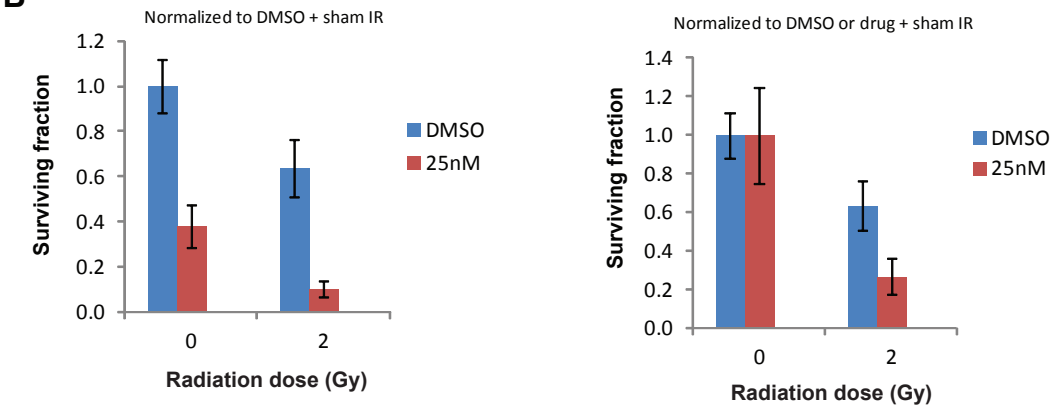

Supplement: Figure S6 — Radiosensitization of RCM-1 cells by AZD7762 (A) and BEZ235 (B) as measured by CFA. (PDF) [file pone.0082982.s006.pdf]

**Figure S7****A****AZD7762****SW837**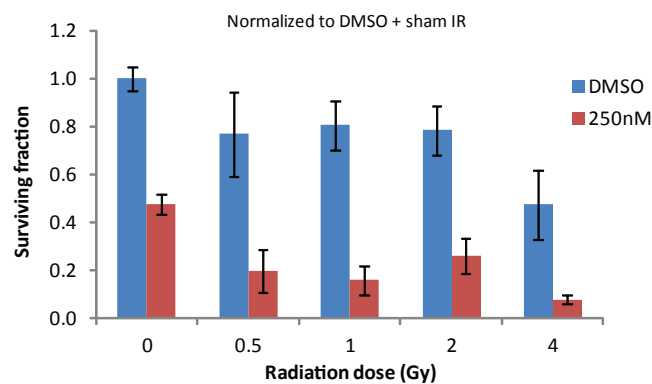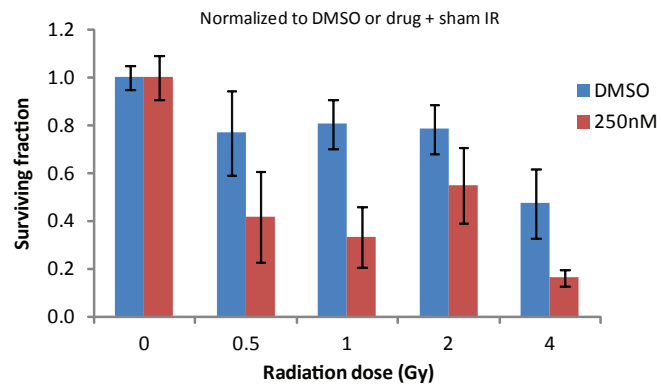**RCM-1**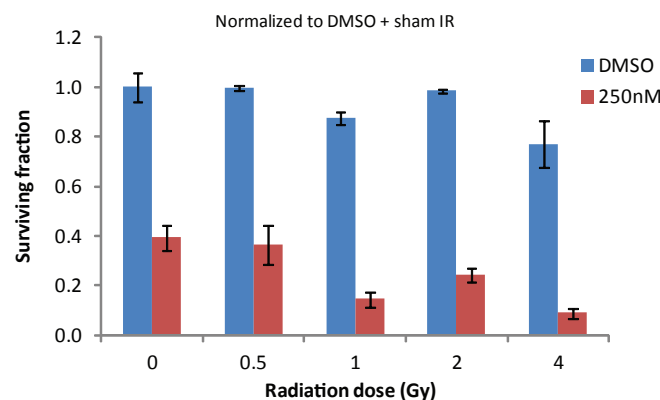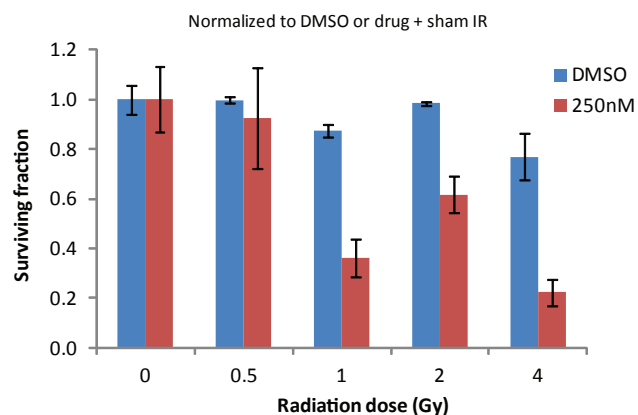**B****BEZ235****SW837**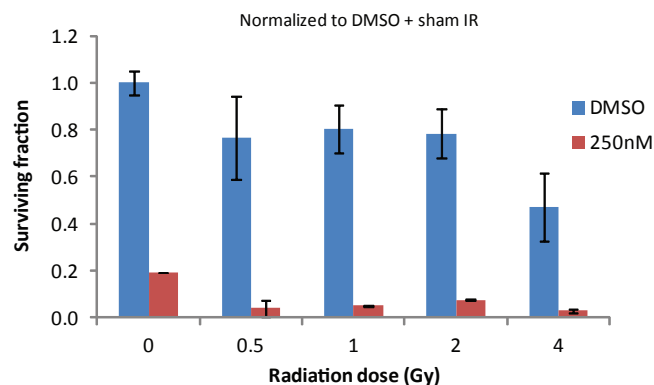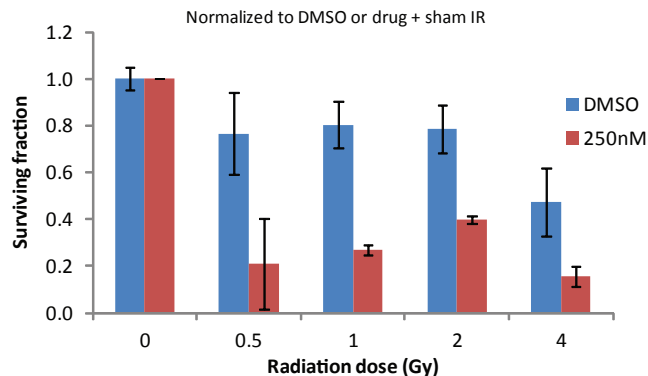**RCM-1**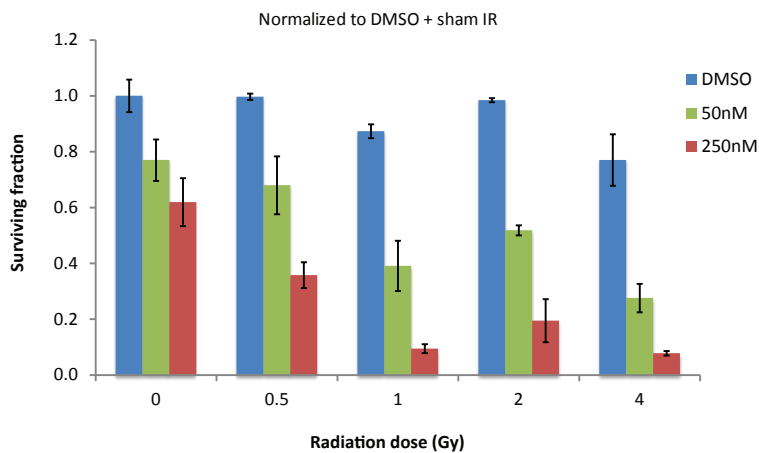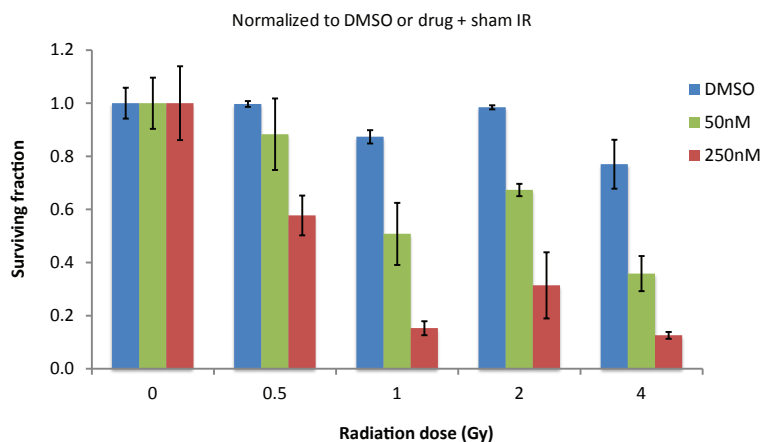

Supplement: Figure S7 — HTA performed with SW837 and RCM-1 cells for AZD7762 (A) or BEZ235 (B) and different doses of IR. (PDF) [file pone.0082982.s007.pdf]

**Figure S8****A**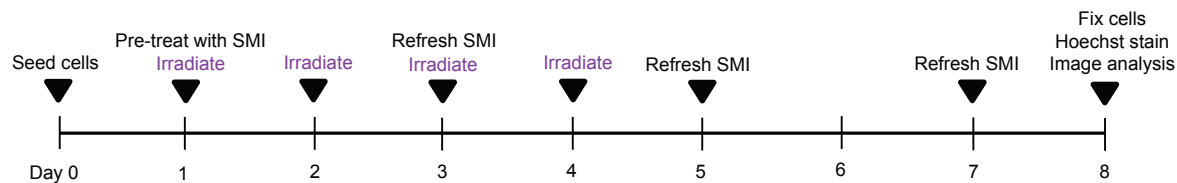**B**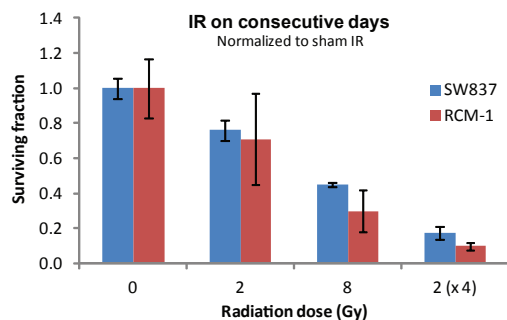**C**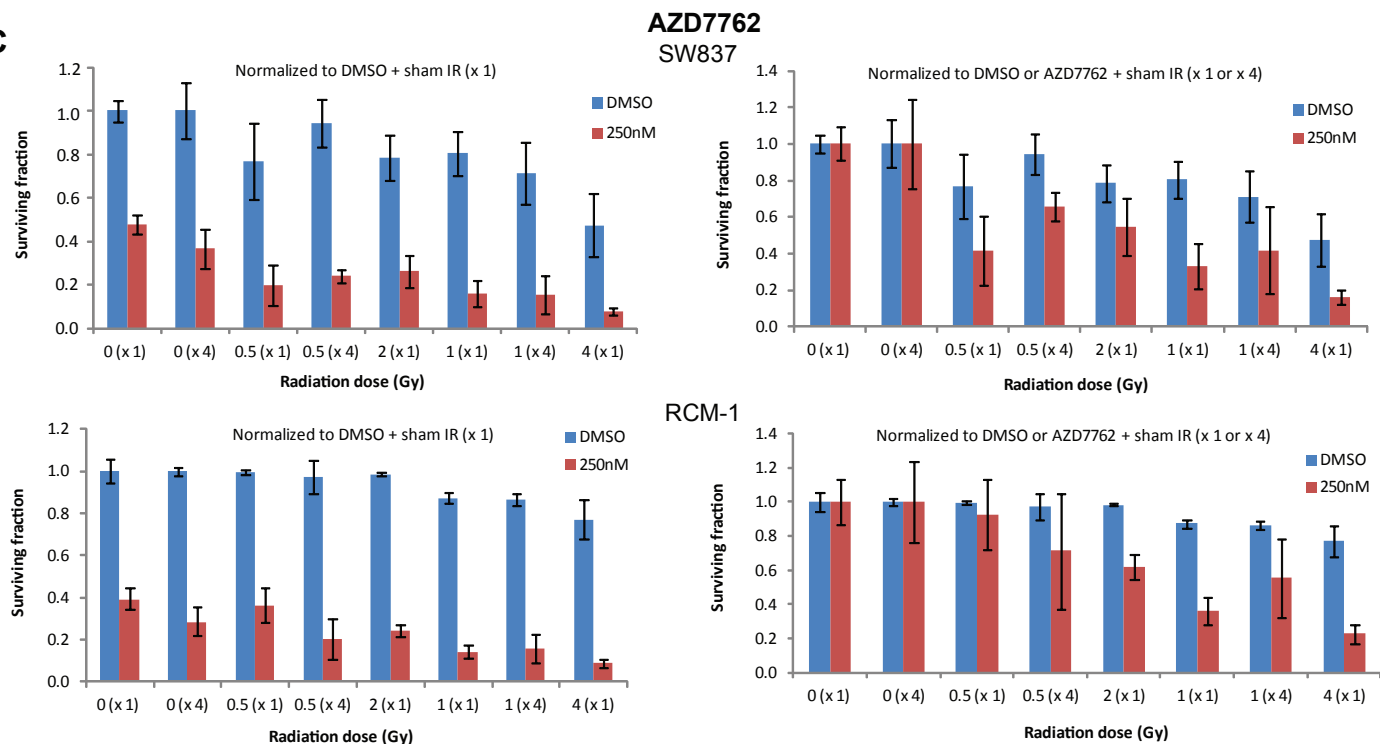**D**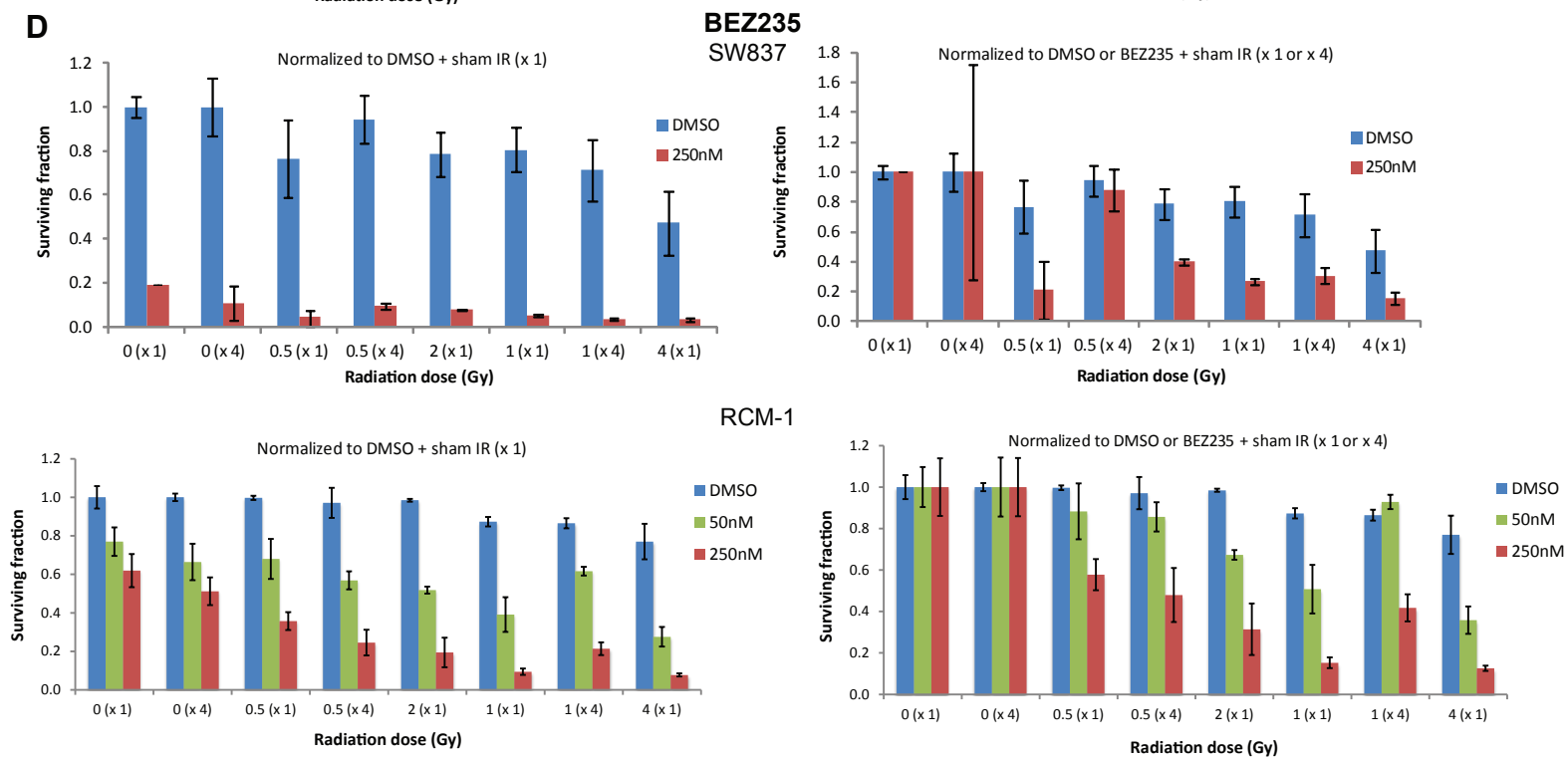

Supplement: Figure S8 — Radiosensitization with IR applied on consecutive days. (A) Modifications to the HTA protocol for applying IR on consecutive days. (B) 2 Gy IR applied on four consecutive days compared to 2 or 8 Gy IR applied once. (C)-(D) 0.5 or 1 Gy IR applied on four consecutive days with or without AZD7762 (C) or BEZ235 (D) compared to various doses of IR applied once. Appropriate sham irradiated controls were included. (PDF) [file pone.0082982.s008.pdf]

**Figure S9**

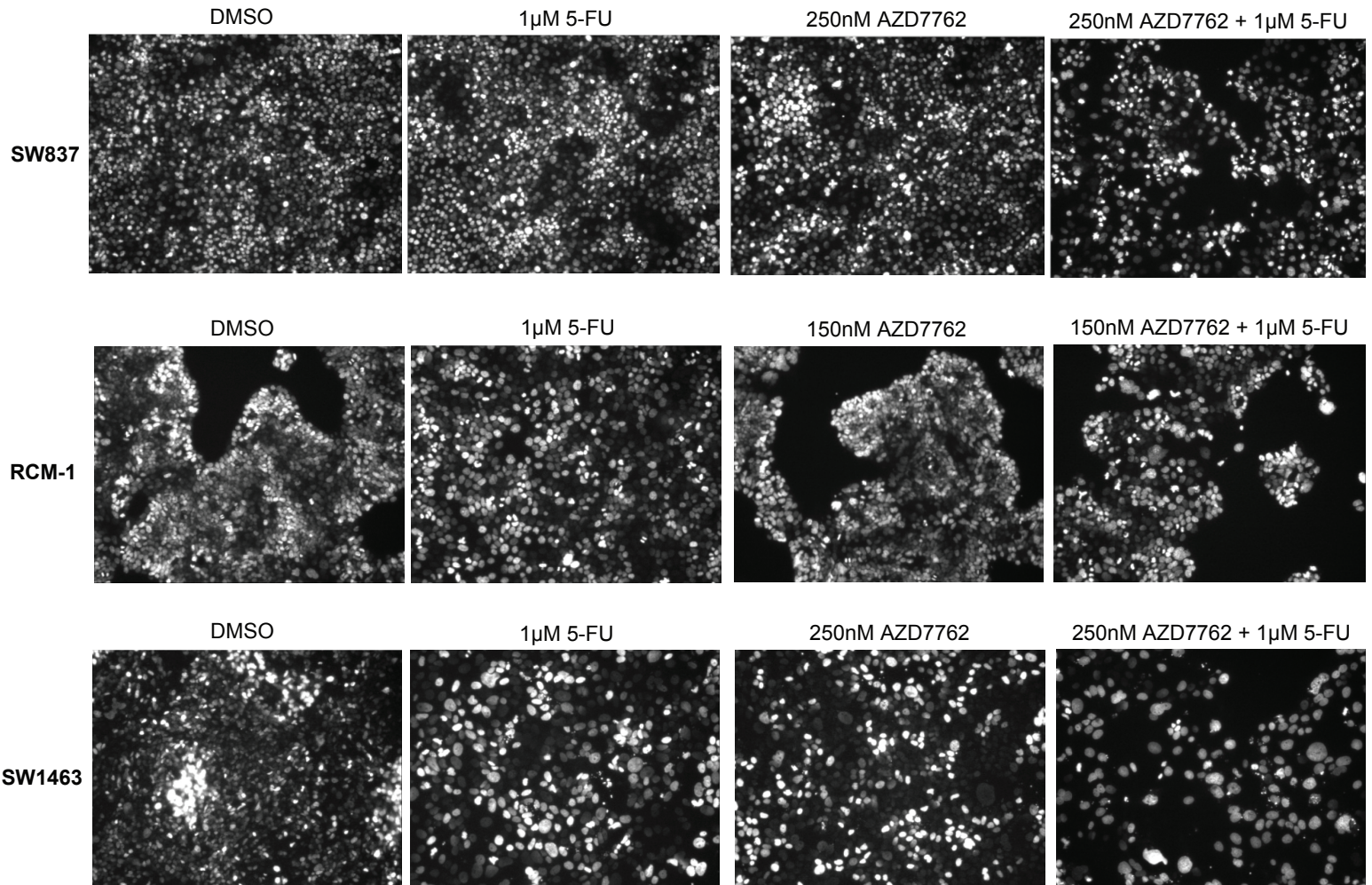

Supplement: Figure S9 — AZD7762 and 5-FU treatments are synergistic. The HTA was performed and nuclei were stained with Hoechst. (PDF) [file pone.0082982.s009.pdf]

Figure S10

A

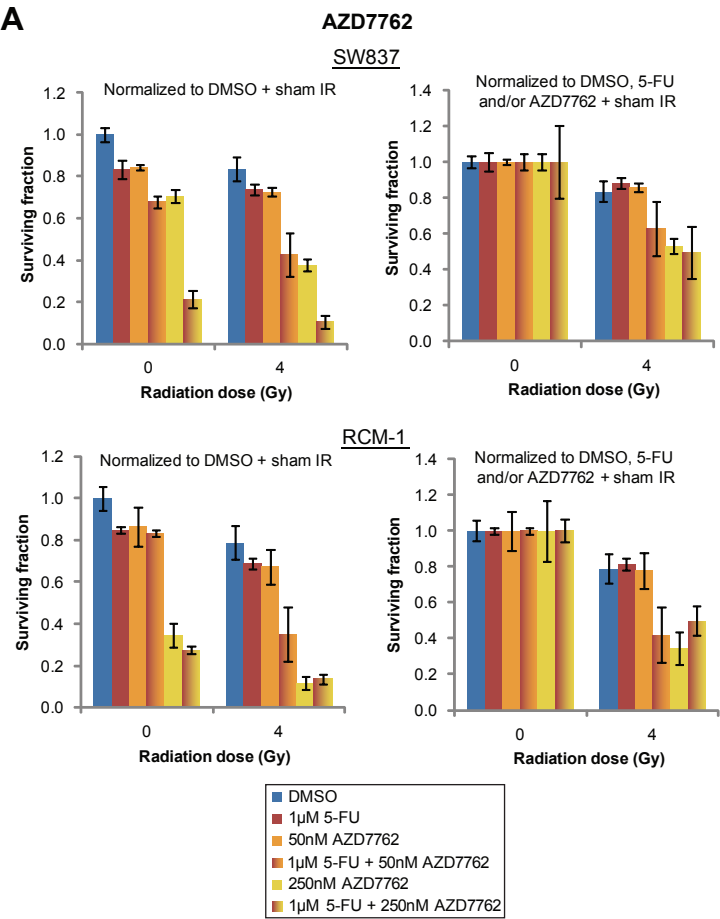

B

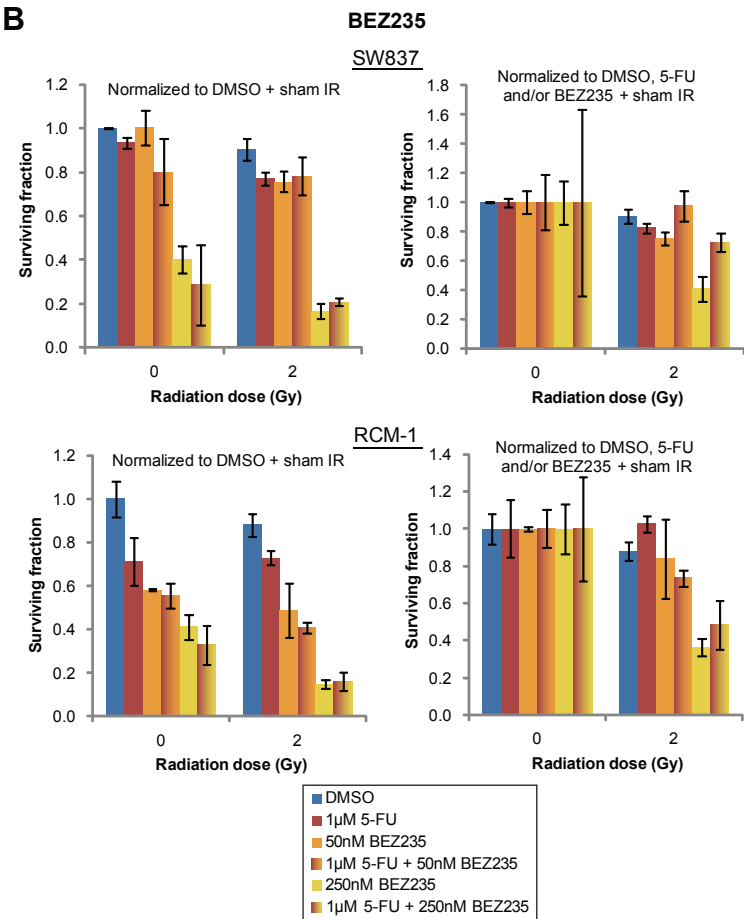

Supplement: Figure S10 — HTA results for the combination of 5-FU, IR and AZD7762 (A) or BEZ235 (B). Synergy between AZD7762 and 5-FU was detected for RCM-1 cells at 150nM AZD7762 (Figure S9), where there is less of an effect of AZD7762 alone. (PDF) [file pone.0082982.s010.pdf]

Figure S11

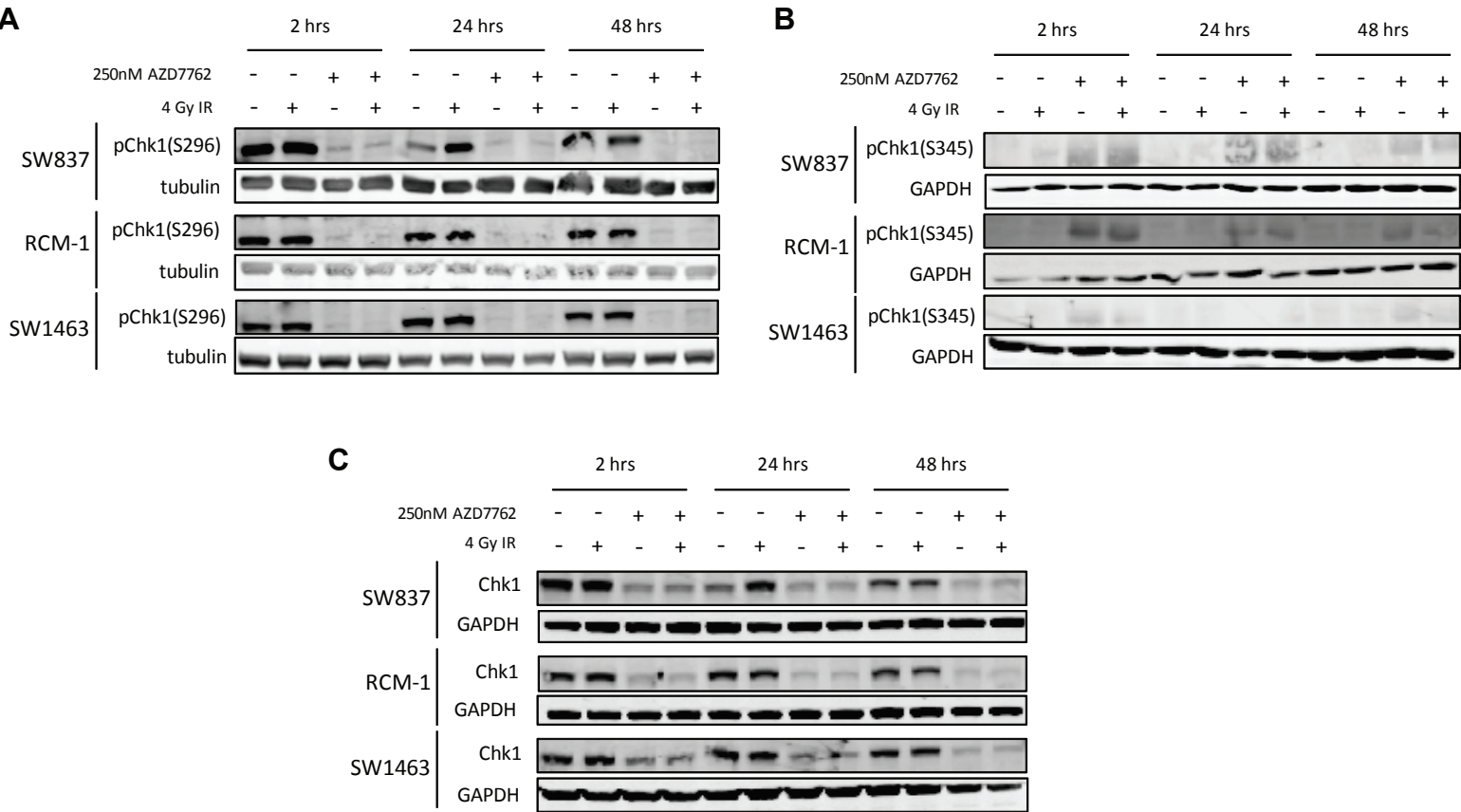

Supplement: Figure S11 — Chk1 is inhibited by AZD7762 in rectal cancer cell lines. The time indicated is post-IR treatment (i.e. cells were exposed to AZD7762 for 4 hours for the “2 hrs” time point). (A) Decreased phosphorylation of a Chk1 autophosphorylation site (S296) by 250nM AZD7762. (B) Increased phosphorylation of Chk1 S345, which is mediated by ATM and ATR and targets Chk1 for degradation. (C) Decreased total Chk1 levels. (PDF) [file pone.0082982.s011.pdf]

Figure S12

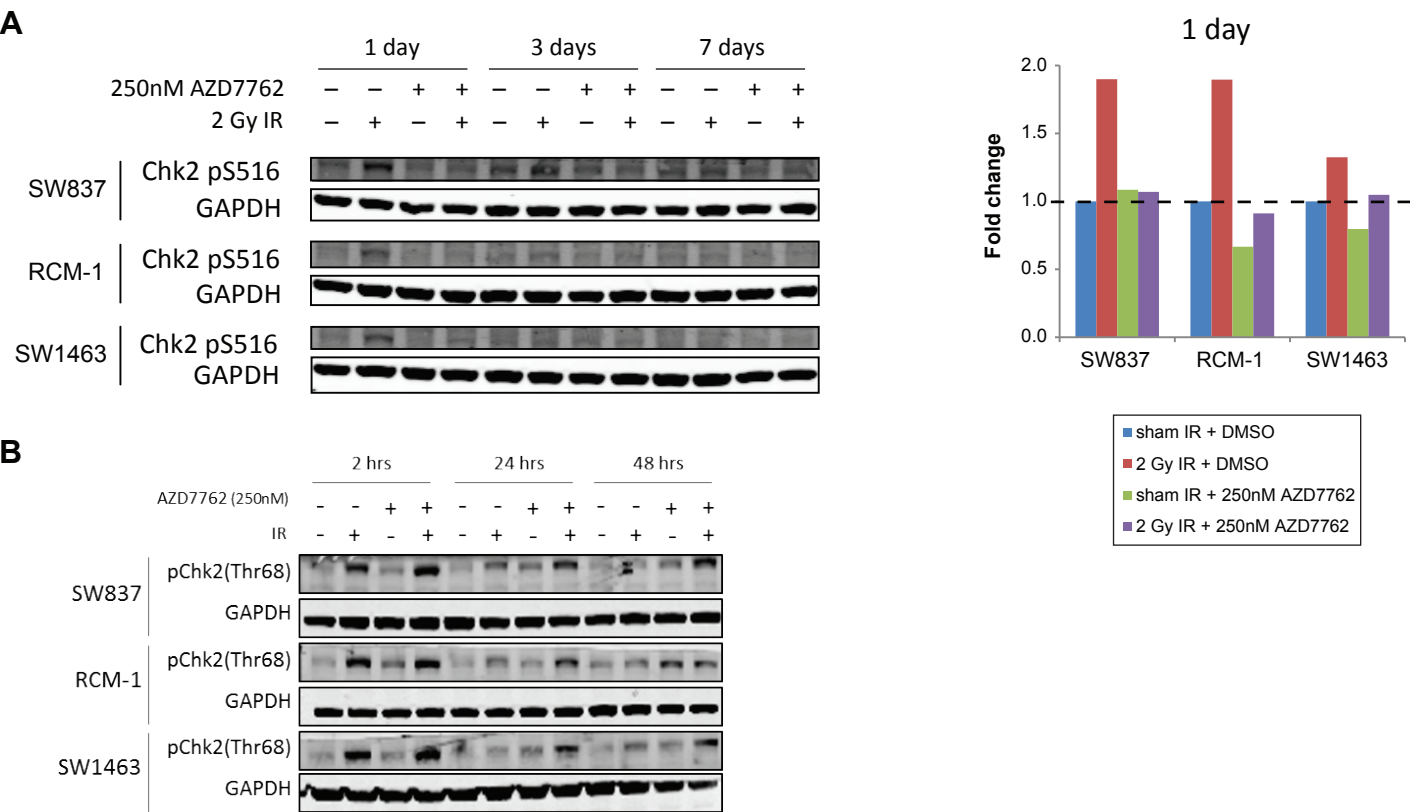

Supplement: Figure S12 — Chk2 is inhibited by AZD7762 in rectal cancer cell lines. The time indicated is post-IR treatment. (A) IR-induced phosphorylation of a Chk2 autophosphorylation site (S516) is inhibited by AZD7762. Right, quantification by background subtraction, normalization to GAPDH, and normalization to control treatment. (B) Phosphorylation of Chk2 site (T68) that is mediated by ATM and ATR. 4 Gy IR was used. (PDF) [file pone.0082982.s012.pdf]

Figure S13

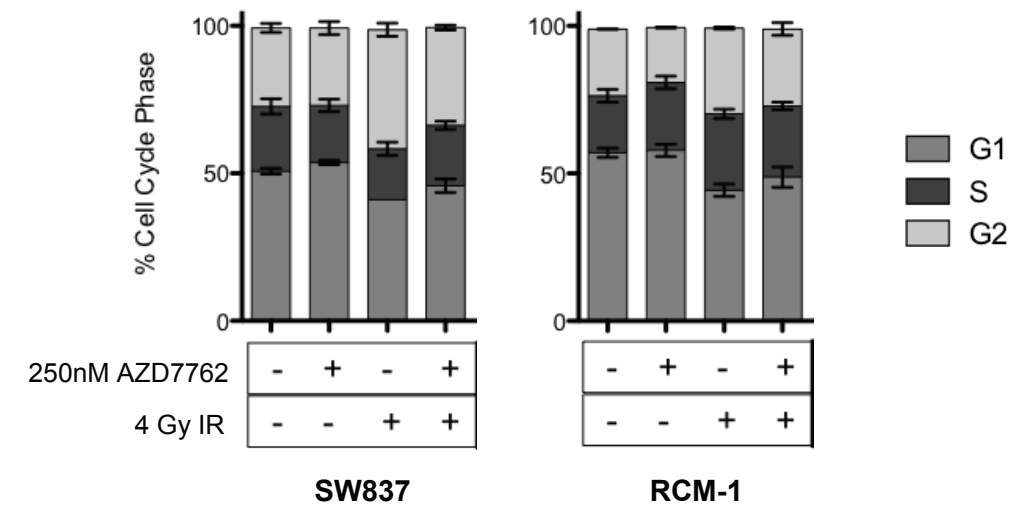

Supplement: Figure S13 — IR-induced G2 arrest is abrogated following treatment with AZD7762. Cell cycle profiling results indicate the percent of cells in different phases of the cell cycle. Cells were treated with AZD7762 for two hours prior to IR, and the analysis was performed after 24 hours. (PDF) [file pone.0082982.s013.pdf]

Figure S14

A

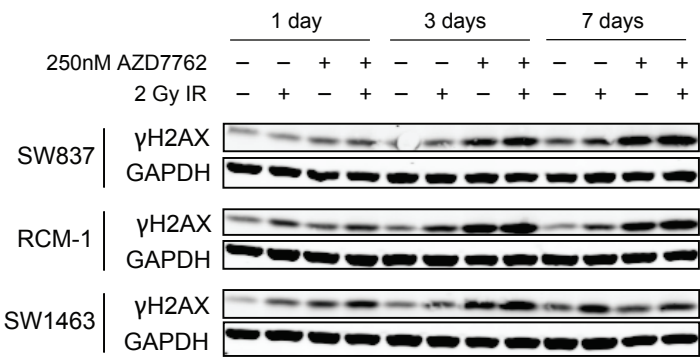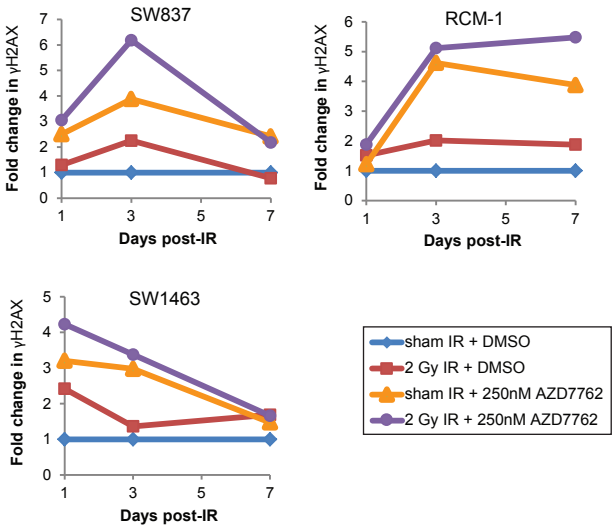

B

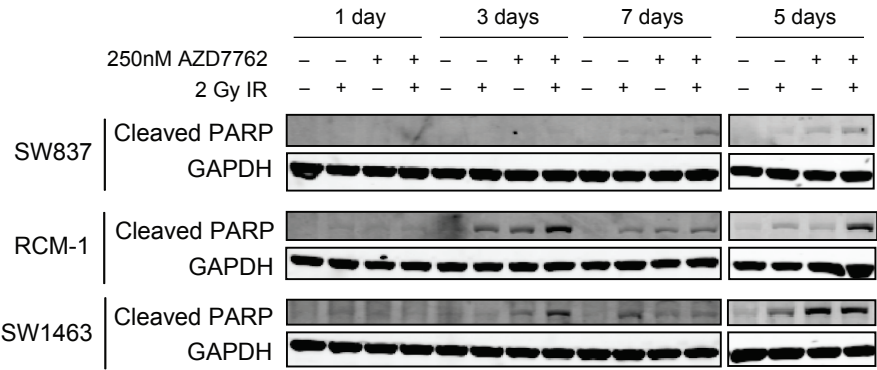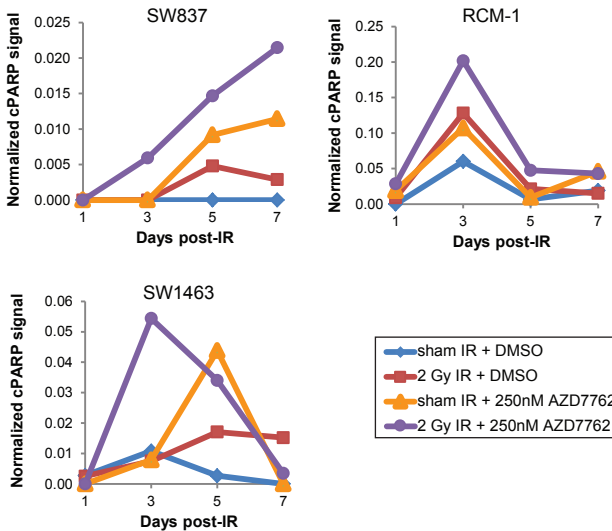

Supplement: Figure S14 — Combination treatment with AZD7762 and IR results in increased DNA damage and induction of apoptosis. (A) DSBs as indicated by γH2AX levels. Right, quantification by background subtraction, normalization to GAPDH, and normalization to control treatment. (B) Apoptosis as indicated by cleaved PARP levels. Right, quantification by background subtraction and normalization to GAPDH. Days post-IR are indicated. (PDF) [file pone.0082982.s014.pdf]

Figure S15

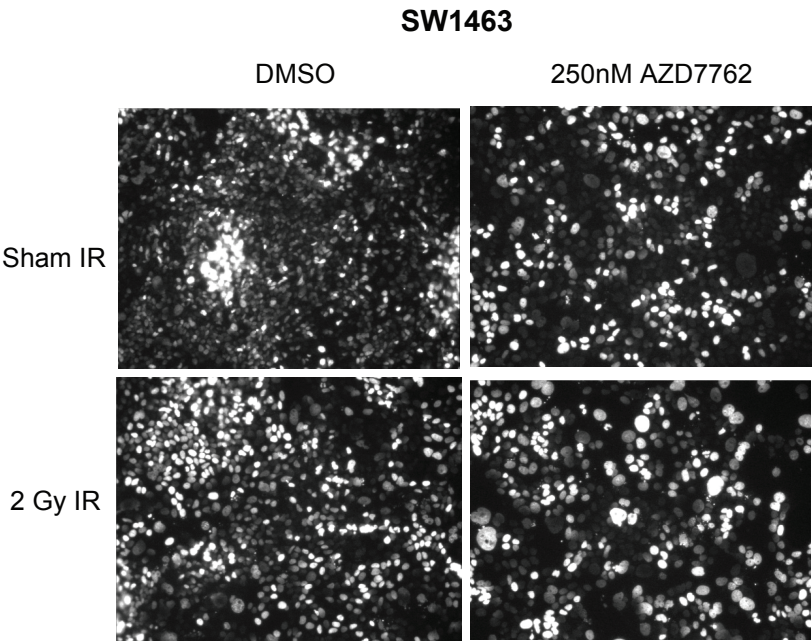

Supplement: Figure S15 — The HTA was performed with SW1463 cells and nuclei were stained with Hoechst. Note that nuclei stain very heterogeneously with Hoechst, especially in the image of AZD7762 treatment alone. (PDF) [file pone.0082982.s015.pdf]
