# Supplementary material for: Comparative Analysis of Radiosensitizers for K-RAS Mutant Rectal Cancers
Source: PLoS One. 2013 Dec 12;8(12):e82982. doi: 10.1371/journal.pone.0082982 (PMC3861465; doi:10.1371/journal.pone.0082982)
Supplement: Table S1 — Mutational status and K-RAS dependency of the human cell lines used in this study. (PDF) [file pone.0082982.s016.pdf]

Table S1. Mutational status and K-RAS dependency of the human cell lines used in this study. *different mutational information has also been published: PANC-1 = K-RAS$^{G12A/+}$ [1], PATU8988T = K-RAS$^{G12V/+}$ [2]. n/a: information not available.

Cell line	Tissue origin	K-RAS	K-RAS dependency	PIK3CA	PTEN	TP53	APC
SW837	Rectum	G12C/+ [3]	Independent [4]	WT [3]	WT [3]	MUT/MUT [3]	MUT/MUT [3]
RCM-1	Rectum	G12V/G12V [3]	Slightly dependent [4]	WT [3]	WT [3]	MUT/+ [3]	WT [3]
SW1463	Rectum	G12C/G12C [3]	Independent [4]	WT [3]	WT [3]	MUT/LOH [3]	MUT/LOH [3]
CaR-1	Rectum	WT [3]	n/a	WT [3]	WT [3]	MUT/MUT [3]	WT [3]
PANC-1	Pancreas	G12D/+ [5] *	Independent [5]	WT [6]	WT [7]	MUT/- [1]	n/a
PATU8902	Pancreas	G12V [5]	Dependent [5]	WT [6]	no protein expressed [6]	MUT [8]	n/a
PATU8988T	Pancreas	G12V/G12V [5] *	Independent [5]	WT [6]	no protein expressed [6]	MUT [8]	n/a
Capan-1	Pancreas	G12V/G12V [3]	Dependent [5]	WT [3]	WT [3]	MUT/MUT [3]	WT [3]
MiaPaca-2	Pancreas	G12C/G12C [3]	n/a	n/a	n/a	MUT/MUT [3]	WT [3]
DLD-1	Colon	G13D/+ [9]	Independent [4]	MUT/+ [10]	WT [11]	MUT/+ [12]	MUT/MUT [13]
HCT116	Colon	G13D/+ [3]	Independent [4]	MUT/+ [3]	WT [3]	WT [3]	WT [3]

1. Butz J, Wickstrom E, Edwards J (2003) Characterization of mutations and loss of heterozygosity of p53 and K-ras2 in pancreatic cancer cell lines by immobilized polymerase chain reaction. BMC Biotechnol 3: 11.
2. Shen YM, Yang XC, Yang C, Shen JK (2008) Enhanced therapeutic effects for human pancreatic cancer by application K-ras and IGF-IR antisense oligodeoxynucleotides. World J Gastroenterol 14: 5176-5185.
3. http://www.sanger.ac.uk/genetics/CGP/cosmic/.
4. Singh A, Sweeney MF, Yu M, Burger A, Greninger P, et al. (2012) TAK1 inhibition promotes apoptosis in KRAS-dependent colon cancers. Cell 148: 639-650.
5. Singh A, Greninger P, Rhodes D, Koopman L, Violette S, et al. (2009) A gene expression signature associated with "K-Ras addiction" reveals regulators of EMT and tumor cell survival. Cancer Cell 15: 489-500.
6. Wallin JJ, Edgar KA, Guan J, Berry M, Prior WW, et al. (2011) GDC-0980 is a novel class I PI3K/mTOR kinase inhibitor with robust activity in cancer models driven by the PI3K pathway. Mol Cancer Ther 10: 2426-2436.

7. Ihle NT, Lemos R, Jr., Wipf P, Yacoub A, Mitchell C, et al. (2009) Mutations in the phosphatidylinositol-3-kinase pathway predict for antitumor activity of the inhibitor PX-866 whereas oncogenic Ras is a dominant predictor for resistance. Cancer Res 69: 143-150.
8. Schumacher G, Kataoka M, Roth JA, Mukhopadhyay T (1999) Potent antitumor activity of 2-methoxyestradiol in human pancreatic cancer cell lines. Clin Cancer Res 5: 493-499.
9. Torrance CJ, Agrawal V, Vogelstein B, Kinzler KW (2001) Use of isogenic human cancer cells for high-throughput screening and drug discovery. Nat Biotechnol 19: 940-945.
10. Samuels Y, Diaz LA, Jr., Schmidt-Kittler O, Cummins JM, Delong L, et al. (2005) Mutant PIK3CA promotes cell growth and invasion of human cancer cells. Cancer Cell 7: 561-573.
11. Lee C, Kim JS, Waldman T (2004) PTEN gene targeting reveals a radiation-induced size checkpoint in human cancer cells. Cancer Res 64: 6906-6914.
12. Sur S, Pagliarini R, Bunz F, Rago C, Diaz LA, Jr., et al. (2009) A panel of isogenic human cancer cells suggests a therapeutic approach for cancers with inactivated p53. Proc Natl Acad Sci U S A 106: 3964-3969.
13. Ilyas M, Tomlinson IP, Rowan A, Pignatelli M, Bodmer WF (1997) Beta-catenin mutations in cell lines established from human colorectal cancers. Proc Natl Acad Sci U S A 94: 10330-10334.
