# Supplementary material for: Comparative Analysis of Radiosensitizers for K-RAS Mutant Rectal Cancers
Source: PLoS One. 2013 Dec 12;8(12):e82982. doi: 10.1371/journal.pone.0082982 (PMC3861465; doi:10.1371/journal.pone.0082982)
Supplement: Table S2 — Drugs used in this study. (PDF) [file pone.0082982.s017.pdf]

Table S2. Drugs used in this study. All SMI were reconstituted with DMSO to a stock concentration of 5mM and stored in aliquots at -20˚C, except for BEZ235 which was stored at a concentration of 2mM and perifosine which was reconstituted in water. 5-FU was reconstituted to a stock concentration of 100mM in DMSO.

Abt888	Selleck Chemicals, Houston, TX
AT-406	Ascenta, Malvern, PA
AUY922	Selleck Chemicals, Houston, TX
AZD1152 -HQPA	Selleck Chemicals, Houston, TX
AZD1480	ChemieTek, Indianapolis IN
AZD6244	ChemieTek, Indianapolis IN
AZD7762	Selleck Chemicals, Houston, TX
AZD8931	Selleck Chemicals, Houston, TX
BEZ235	LC Laboratories, Woburn, MA
BKM120	Selleck Chemicals, Houston, TX
CI1040	LC Laboratories, Woburn, MA
Dovitinib	LC Laboratories, Woburn, MA
GDC-0941	LC Laboratories, Woburn, MA
GDC-0980	ChemieTek, Indianapolis IN
Gefitinib	LC Laboratories, Woburn, MA
GSK1120212	ChemieTek, Indianapolis IN
KU-55933	Selleck Chemicals, Houston, TX
LY2228820	Selleck Chemicals, Houston, TX
Midostaurin	LC Laboratories, Woburn, MA
PD-0332991	ChemieTek, Indianapolis IN
PD0325901	ChemieTek, Indianapolis IN
Perifosine	LC Laboratories, Woburn, MA
PLX4032	LC Laboratories, Woburn, MA
Raf265	Selleck Chemicals, Houston, TX
Sorafenib	LC Laboratories, Woburn, MA
SP600125	EMD Millipore, Billerica, MA
Sunitinib	LC Laboratories, Woburn, MA
Vorinostat	ChemieTek, Indianapolis IN
5-FU	Sigma-Aldrich, St. Louis, MO
SCH900776	ChemieTek, Indianapolis IN
LY2603618	Selleck Chemicals, Houston, TX
