# Supplementary material for: Comparative Analysis of Radiosensitizers for K-RAS Mutant Rectal Cancers
Source: PLoS One. 2013 Dec 12;8(12):e82982. doi: 10.1371/journal.pone.0082982 (PMC3861465; doi:10.1371/journal.pone.0082982)
Supplement: Table S3 — Antibodies used in this study. (PDF) [file pone.0082982.s018.pdf]

Table S3. Antibodies used in this study.

**Specificity**	**Dilution**	**Catalog #**	**Manufacturer**
Chk1	1:1000	2345	Cell Signaling, Danvers, MA
Cleaved PARP	1:1000	9546	Cell Signaling, Danvers, MA
GAPDH	1:10000	Ab8245	Abcam, Cambridge, MA
Phospho-Chk1 (Ser296)	1:1000	2349	Cell Signaling, Danvers, MA
Phospho-Chk1 (Ser345)	1:250	2348	Cell Signaling, Danvers, MA
Phospho-Chk2 (Thr68)	1:1000	2661	Cell Signaling, Danvers, MA
γH2AX	1:1000	9718	Cell Signaling, Danvers, MA
β-tubulin	1:10000	T6074	Sigma-Aldrich, St. Louis, MO
Phospho-Chk2 (Ser516)	1:1000	2669	Cell Signaling, Danvers, MA
